# Temperature Anomalies and Structural Change in Global and Mediterranean Apiculture Systems

**DOI:** 10.3390/insects17080867

**Published:** 2026-08-20

**Authors:** Okan Özgül, Rahşan İvgin Tunca, Gonca Özmen Özbakır

**Affiliations:** 1 Beekeeping Program, Department of Crop and Animal Production, Ula Ali Koçman Vocational School, Muğla Sıtkı Koçman University, Ula, Muğla 48640, Türkiye; 2 Department of Animal Science, Faculty of Agriculture, Harran University, Şanlıurfa 63050, Türkiye; gozmenozbakr@harran.edu.tr

**Keywords:** temperature anomalies, apiculture systems, honey production value, colony stocks, Mediterranean basin, long-term time series

## Abstract

While climate change is a significant pressure point for pollinators and honeybees, its long-term relationship with managed honeybee colonies and honey production remains unclear and context sensitive. This study, using FAOSTAT data from 1961 to 2024, examined the relationships between land surface temperature anomalies and the number of registered beehives, the constant-price honey production value, and the normalized honey production value per colony, both globally and specifically for 18 Mediterranean countries. To differentiate between long-term structural trends and short-term temperature relationships, correlation, regression, bootstrap confidence interval, temperature series sensitivity analysis, cross-correlation function, and normalized index analyses were used together. The study results show strong long-term co-movement between temperature anomalies and recorded beekeeping indicators at the global and Mediterranean scale in the level series; however, first-differenced (year-to-year) analyses show this association weakens substantially and, for Mediterranean honey production value, reverses sign, indicating that the level-series correlations are driven largely by common long-term trends. The relationship between temperature anomalies and beekeeping indicators shows a context-dependent structure among Mediterranean countries. The findings show that the increase in colony size does not always coincide with the increase in the constant-price honey production value per colony; in some cases, these two indicators can move in different directions. This indicates the need for an analytical decomposition of the dynamics of quantitative growth and economic production value per colony in the beekeeping sector.

## Simple Summary

Rising temperatures, changes in rainfall patterns, and the increase in extreme weather events directly affect biological systems, agricultural ecosystems, and pollinators such as honeybees. Honeybees are an important element of the ecological system. Besides their contribution to pollination, their valuable beekeeping products also make them economically indispensable. Our aim is to reveal the statistical relationships between temperature anomalies and recorded beekeeping indicators at both the global and Mediterranean basin scales. In this study, the dataset covers the years 1961–2024 and consists of three main variables: land surface temperature anomaly (°C), number of bee colonies (units), and honey production value at constant prices. The results indicate long-term covariation between temperature anomalies and recorded beekeeping indicators, together with substantial heterogeneity among Mediterranean countries; the strength and even the direction of this covariation differ between the level series and year-to-year changes, particularly for honey production value. At the same time, the normalized index of honey production value per colony revealed that the dynamics of economic production value per colony do not always progress in parallel with the increase in the number of colonies. The study shows that the relationships between temperature anomalies and beekeeping indicators cannot be evaluated within a one-dimensional framework; it is a multi-layered process in which long-term structural expansion and cross-country heterogeneity are both evident, though the current data cannot establish how these two dimensions interact.

## 1. Introduction

Climate change constitutes a multifaceted pressure mechanism that transforms biological production systems not only through increasing average temperatures, but also through changes in precipitation patterns, heat waves, increased frequency of droughts, and phenological shifts [1,2,3,4]. This transformation is of particular importance for the beekeeping sector, which is highly dependent on climate and ecosystem interactions. Honeybees (*Apis mellifera*) play a central role both in the production of honey and other hive products, and in pollination services, which are critical for the sustainability of agricultural production and natural ecosystems [5]. Since a significant portion of global food systems depend on pollinators, climate induced changes in bee populations affect not only the beekeeping sector but also broader agroecological resilience [5,6].

While the association between climate change and honeybees has long been studied, there is no complete consensus in the literature regarding the direction and magnitude of this association. On the one hand, it has been shown that rising temperatures, drought, floral resource depletion, and plant-pollinator phenological incompatibility can lead to colony stress, food reserve loss, and production decline [7,8,9]. Particularly in climate change sensitive regions like the Mediterranean, it has been reported that high temperatures and drought conditions can shorten flowering periods, suppressing colony development and honey production [8]. In addition, recent continental scale analyses reveal that climate change is increasing pressure on bee food sources. In this regard, increasing temperatures and drought conditions are limiting the available food sources for bees, deepening seasonal food deficits. Rising temperatures and decreasing rainfall are reducing the diversity of food sources available to bees; furthermore, climate pressures can weaken the resilience of plant pollinator interaction networks. Therefore, climate change is related not only to the level of production but also to the structure of the ecological networks on which bees feed [10].

It is important to note that colony stress, floral-resource depletion, and phenological mismatches are mechanisms documented in the cited literature through direct biological, ecological, or experimental measurements; none of these mechanisms is measured directly in the present study. The dataset analysed here consists of registered colony numbers (a sectoral/administrative stock indicator) and constant-price honey production value (an economic indicator), rather than direct measures of colony survival, population dynamics, or physical honey yield. Accordingly, the present study should be understood as an analysis of recorded beekeeping-sector indicators in relation to temperature anomalies, not as a direct test of the biological mechanisms reviewed above.

On the other hand, the relationship between climate change and beekeeping is too complex to be explained solely through negative impacts. Some long-term studies have shown that moderate warming under certain agroecological conditions may be associated with longer production seasons, altered harvest phenology, and increased honey yields [11]. Similarly, recent systematic reviews highlight that climate change can have negative, neutral, and context-dependent positive effects on honeybees, thus emphasizing that the relationship is not linear and unidirectional [12]. These studies suggest that the relationship between climate change and beekeeping cannot be explained by a dichotomy of “collapse” or “benefit”; rather, it is a heterogeneous association depending on scale, region, the period of time, production indicators, and methods used. Despite this heterogeneity, two key gaps stand out in the current literature. First, the majority of studies focus on short-term climate events, experimental stress responses, or colony health indicators; in contrast, long-term analyses that consider colony size and production dynamics together at the macro scale, spanning decades, remain quite limited [12]. Secondly, most previous studies have focused on overall measures such as total colony numbers or total honey production. In contrast, relatively few studies have examined the difference between colony size and production performance at the colony level. Evidence from long-term datasets suggests that this distinction is important. It also indicates that productivity is influenced by climate, environmental conditions, and colony dynamics through different pathways [13,14]. This knowledge gap is particularly pronounced in the Mediterranean basin. The Mediterranean serves as a critical case for studying the association between climate change and beekeeping given warming above the global average, increased hydroclimatic stress, and high biogeographic vulnerability [2]. However, studies conducted in this region have mostly remained local or short-term, with limited long-term comparative analyses at the basin scale. Furthermore, recent European scale studies indicate that the impacts of climate change on beekeeping are felt more severely in the Mediterranean and Southern Europe, while adaptation capacity shows regional differences [15]. This suggests that the relationship between temperature anomalies and beekeeping indicators may not be homogeneous; it may exhibit an asymmetrical structure depending on country-specific thresholds, production structure, and adaptation dynamics.

Climate change is a multidimensional process encompassing changes in precipitation, drought frequency, heat waves, and phenological shifts, in addition to rising mean temperatures. The present analysis is restricted to annual land surface temperature anomaly, the only climatic variable available at the required country-year resolution and time span (1961–2024) in the FAOSTAT database. Other dimensions of climate change—particularly precipitation and drought, which are likely closely linked to floral resource availability in the Mediterranean—were not available at comparable coverage and are therefore not included. Consequently, temperature anomaly is used here partly as a broad temporal indicator of overall warming during the study period, rather than as an isolated explanatory climatic factor capturing all dimensions of climate change relevant to beekeeping.

Three beekeeping indicators are analysed throughout this study and are defined here to avoid ambiguity. Registered colony number is a sectoral stock indicator reflecting official beekeeping capacity rather than a direct biological population count. Constant-price honey production value is an economic indicator (in international dollars) reflecting the value of production adjusted for inflation and exchange-rate effects, not physical honey yield in kilograms. The normalized honey production value per colony index is a derived, country-specific relative measure expressing the change in the ratio of honey production value to colony number, relative to each country’s own baseline; it does not measure biological productivity or honey yield per colony. These distinctions are elaborated further in Section 2.

Long-term changes in FAOSTAT colony numbers and production values may reflect not only climatic conditions but also changes in registration coverage, national reporting procedures, beekeeping policy, market structure, management practices, disease pressure, technology, and sectoral organization over more than six decades. These factors are treated here as central to the interpretation of observed trends, rather than as secondary caveats, and motivate the descriptive, association-based framing of the research question adopted in this study.

This study aims to address this gap. Using FAOSTAT data from 1961 to 2024, this study examines long-term and short-term statistical associations between temperature anomalies recorded beekeeping indicators—registered bee colony number, constant-price honey production value, and the normalized honey production value per colony index—comparing their trajectories at the global scale and specifically across 18 Mediterranean countries, and evaluating heterogeneity among these countries. The study offers three original contributions. First, it provides a long-term macro perspective spanning over sixty years, going beyond the existing short-term literature. Second, it evaluates structural heterogeneity in these associations by analyzing colony-number growth and economic production value per colony as distinct sector-level indicators. Third, the study examines whether the associations between temperature anomalies and beekeeping indicators are similar across countries. The study does not measure adaptation, vulnerability, colony loss, or causal climatic effects, as these concepts are not operationalized with the variables available here; rather, it contributes to understanding how recorded beekeeping-sector indicators have co-evolved with temperature anomalies over six decades, and how strongly their trajectories differ among Mediterranean countries.

## 2. Materials and Methods

### 2.1. Data Source and Scope

All data used in the analysis were obtained from FAOSTAT, the open data platform of the Food and Agriculture Organization of the United Nations (FAO). The dataset covers the years 1961–2024 and consists of three main variables: land surface temperature anomaly (°C), number of bee colonies (units), and honey production value at constant prices. Since FAOSTAT data are based on annual reports from countries, coverage varies across countries and years; the resulting panel structure and its consequences for the basin-level series are described in Section 2.2. Interpolation or data completion was not applied for missing years, and analyses were carried out solely based on data available at FAOSTAT.

The geographical scope is defined in two layers: global scale (World) and Mediterranean basin scale. For the Mediterranean basin, 18 countries are included: Albania, Algeria, Bosnia and Herzegovina, Croatia, Egypt, France, Greece, Israel, Italy, Lebanon, Libya, Montenegro, Morocco, Slovenia, Spain, Syria, Tunisia, and Türkiye. The 18 countries were selected as those with a Mediterranean coastline that are represented as separate reporting units in FAOSTAT for all three analysed variables throughout the study period. Cyprus was not included because its beekeeping statistics are reported under separate administrative entities, which prevents construction of a single consistent country-level series for the full period. Malta was not included because FAOSTAT reports no colony data for this country. Türkiye is included in the analysis both as a basin whole and as a separate observation unit in the country-based panel analysis. The temperature variable was defined as the FAOSTAT Element code Temperature change, the colony number as the Stocks element, and the honey production value as the Gross Production Value constant 2014–2016 thousand Int$ element. All data were extracted from FAOSTAT on 10 April 2026. Temperature anomaly was taken from the Temperature change on land domain (domain code ET, element code 7271, meteorological year, months code 7020); colony number from the Crops and livestock products domain (domain code QCL, element code 5114 Stocks, item Bees, CPC item code 02196); and honey production value from the Value of Agricultural Production domain (domain code QV, element code 152, item Natural honey, CPC item code 02910) [16,17,18]. In this study, climate change was represented through the land surface temperature anomaly indicator in the FAOSTAT dataset. Other environmental and human factors such as rainfall, drought, extreme temperature events, land use, pesticide pressure, diseases, beekeeping management, and socioeconomic support were not included in the model. Therefore, the analysis results were interpreted as statistical relationships between temperature anomalies and recorded beekeeping indicators, rather than as a direct effect of all components of climate change. The colony number variable was considered not as a direct measure of biological colony reproduction, but as a stock indicator reflecting the registered beekeeping capacity and sector scale of countries. Hence, long-term increases in colony numbers were interpreted not only in relation to climatic conditions, but also in conjunction with beekeeping policies, producer behaviors, economic incentives, changes in registration systems, and expansion in commercial beekeeping capacity. The use of a fixed rate international dollar (Int$) allows for the comparison of honey production values of different countries, adjusted for inflation and exchange rate effects.

### 2.2. Data Merging and Derived Variables

World dataset: Temperature, colony, and honey files were combined into a single table via a merging procedure (inner join) using the year as the merge key. As a result, *n* = 64 observations were obtained (1961–2024).

Mediterranean panel dataset: The same merging procedure was performed using country and year as a composite merge key, yielding a total of 956 country-year observations for the 18 Mediterranean countries included in the analysis. The panel dataset is unbalanced because the number of observations available for each country is not the same. The number of observations per country varies between 19 and 64 years depending on data availability. The shortest series belongs to Montenegro with 19 observations; Türkiye, Egypt, Israel, and some other countries have 64 observations covering the entire period 1961–2024. Because the dataset has an unbalanced panel structure, each country was analysed using only the years for which data were available. Missing observations were not interpolated or otherwise completed. This was particularly relevant for honey production value, which was unavailable after 2017 for six countries (Croatia, France, Greece, Italy, Slovenia, Spain), reducing the basin composition for honey-related analyses from 18 to 12 countries from 2018 onward. Colony-number data remained complete for all 18 countries through 2023, with a corresponding reduction only in 2024. Full country-year coverage details are reported in Supplementary Table S2a,b. As a result, all analyses were based solely on observed FAOSTAT records, and no artificial data were introduced into the dataset. Consequently, two analytical tracks were used: a colony-based track (temperature and colony number), covering 1961–2023 with all 18 countries, and a honey-based track (temperature, colony number, and honey production value), covering 1961–2017 with all 18 countries. World-level series were unaffected, as they represent FAOSTAT’s pre-aggregated global totals rather than an in-house merge across countries.

Mediterranean annual total/average and temperature sensitivity analysis: To create a basin-level time series, colony numbers and honey production values were collected annually, while the temperature anomaly was represented by taking the equally weighted arithmetic mean of the 18 Mediterranean countries included in the analysis. In this approach, each country was considered an observation unit regardless of its area, population size, or beekeeping capacity. Therefore, the Mediterranean temperature indicator used in the main analyses is not an area-weighted physical Mediterranean climate average, but a country-based regional temperature anomaly indicator. The basic variables used at the basin level were calculated using Equation (1) for temperature anomaly, Equation (2) for colony number and Equation (3) for honey production value.

(1) $$Temp_{basin}\left(t\right)=\frac{1}{\left|S_{t}\right|}\sum_{i\in S_{t}}Temp_{i}\left(t\right)$$

(2) $$Colony_{basin}\left(t\right)=\sum_{i\in S_{t}}Colony_{i}\left(t\right)$$

(3) $$Honey_{basin}\left(t\right)=\sum_{i\in S_{t}}Honey_{i}\left(t\right)$$

In equations, Temp_i(t) represents the temperature anomaly value for country i in year t; Colony_i(t) represents the number of colonies in country i in year t; and Honey_i(t) represents the constant-price honey production value for country i in year t. Accordingly, Temp_basin(t) represents the equally weighted average temperature anomaly across the countries reporting in year t; Colony_basin(t) and Honey_basin(t) represent the total number of colonies and the total honey production value at the basin level, respectively. S_t denotes the set of countries reporting in year t, and |S_t| the number of such countries.

Equally weighted national averages used in the main analysis. However, an additional sensitivity analysis was conducted to assess the potential impact of the Mediterranean temperature indicator calculation method on the results. In this context, in addition to the equally weighted temperature series, an area-weighted temperature series based on national surface area and a colony-weighted temperature series based on annual colony count were calculated. The area-weighted and colony-weighted series were not used in place of the main analyses; they were considered as supplementary analyses to evaluate the sensitivity of the results to the choice of temperature indicator. The alternative temperature series used in the sensitivity analysis were calculated using Equations (4) and (5) for area-weighted temperature and Equations (6) and (7) for colony-weighted temperature.

(4) $$Temp_{area}\left(t\right)=\sum_{i\in S_{t}}\left[w_{area,i}\left(t\right)\times Temp_{i}\left(t\right)\right]$$

(5) $$w_{area,i}\left(t\right)=\frac{Area_{i}}{\sum_{j\in S_{t}}Area_{j}}$$

(6) $$Temp_{colony}\left(t\right)=\sum_{i\in S_{t}}\left[w_{colony,i}\left(t\right)\times Temp_{i}\left(t\right)\right]$$

(7) $$w_{colony,i}\left(t\right)=\frac{Colony_{i}\left(t\right)}{\sum_{j\in S_{t}}Colony_{j}\left(t\right)}$$

The number of countries contributing to the basin-level series varies by year, increasing from 13 in 1961 to 18 from the mid-2000s onward, and the series are therefore computed over the countries reporting in each year, with the weights in Equations (5) and (7) renormalized accordingly (Supplementary Table S2b). The colony-based series are reported to 2023 and the honey-based series to 2017, since honey production value is unavailable for six countries thereafter. Because the indices are anchored to 1961, when 13 countries reported, part of the apparent long-term increase reflects the entry of additional reporting countries rather than change within countries.

In the equations, Area_i represents the surface area of country i; w_area,i(t) represents the share of country i in the total surface area of the countries reporting in year t; and w_colony,i(t) represents the share of the number of colonies in country i in year t in the total number of colonies in the basin in the same year. The weights are renormalized over S_t each year. Thus, the stability of the main findings against the temperature weighting method was evaluated by comparing equally weighted, area-weighted, and colony-weighted temperature series.

### 2.3. Normalization Procedures

Two different normalization methods were applied in the analysis, and it is critical to keep them separate.

#### 2.3.1. Time Series Index

Colony number and honey production value were indexed based on the year 1961 for visual comparison purposes. This process allows both variables to be tracked independently of different units on the same graph; the indexing process was carried out with Equation (8).

Index(t) = [X(t)/X(1961)] × 100(8)

For example, the number of colonies worldwide was 49,172,039 in 1961, and this value was taken as 100; the expected increase to 101,713,116 in 2024 gave an Index ≈ 207. This normalization was performed for visualization purposes only and was not used in correlation or regression calculations.

#### 2.3.2. Normalization of Honey Production Value Index per Colony

For each country, the honey production value per colony was first calculated as an indicator of raw economic production value using Equation (9) and then subjected to intra-country normalization using Equation (10). The index used here does not directly represent the physical honey yield, i.e., honey production in kilograms per colony. The index was evaluated as an indicator of economic production value per colony, which expresses the ratio of the constant-price honey production value to the number of colonies.

As a sensitivity check, the index was also computed using a 3-year baseline mean (Equation (11)) instead of the single starting-year value.

(9) $$Efficiency_{i}\left(t\right)=\left[\frac{Honey_{i}\left(t\right)}{Colony_{i}\left(t\right)}\right]\times 1000$$

(10) $$EffNorm_{i}\left(t\right)=\frac{Efficiency_{i}\left(t\right)}{Efficiency_{i}\left(t_{0}\right)}$$

(11) $$EffNorm_{i}\left(t\right)=\frac{Efficiency_{i}\left(t\right)}{\frac{1}{3}\sum_{j=0}^{2}Efficiency_{i}\left(t_{0}+j\right)}$$

Here, Efficiency_i(t) represents the constant-price honey production value per 1000 colonies in country i in year t; Honey_i(t) represents the constant-price honey production value in country i in year t; Colony_i(t) represents the number of registered colonies in country i in year t; EffNorm_i(t) represents the normalized honey production value index per colony; and t_0_ represents the first year in the data series for each country, usually 1961.

Consequently, the starting value for each country was set at 1.0, and temporal variation was tracked based on this baseline. Therefore, the index shows the relative change in the economic value of honey production per colony within a country, not the biological honey productivity. An increase in the index indicates that the constant-price honey production value per colony in that country has increased compared to the starting year; a decrease indicates that this indicator has declined compared to the starting year. The purpose of this normalization is as follows: Türkiye starts with 180 Int$/1000 colonies per unit, while Albania has a different starting point. Comparison based on raw values leads to large countries suppressing smaller countries. Normalization eliminates this scale pressure and allows each country to represent the variation within itself. For the basin average, the arithmetic mean of the normalized honey production value per colony indices of the existing countries is taken each year, and this process is shown by Equation (12).

(12) $$EffNorm_{basin}\left(t\right)=\frac{1}{N_{t}}\sum_{i=1}^{N_{t}}EffNorm_{i}\left(t\right)$$

Within the reported range, the time-varying denominator N_t is equal to 18 in all years.

### 2.4. Correlation Analysis

#### 2.4.1. Pearson Correlation Coefficient

The Pearson correlation coefficient was used to evaluate the direction and strength of the linear relationship between two continuous variables. In this study, the linear relationships between temperature anomaly and colony number, honey production value and normalized honey production value per colony index were calculated using Equation (13).

(13) $$r=\frac{\sum \left[\left(X_{i}-\bar{X}\right)\left(Y_{i}-\bar{Y}\right)\right]}{\sqrt{\sum {\left(X_{i}-\bar{X}\right)}^{2}\times \sum {\left(Y_{i}-\bar{Y}\right)}^{2}}}$$

Here, r represents the Pearson correlation coefficient; X_i_ and Y_i_ represent the values of the relevant variables in observation i; and $\bar{X}$ and $\bar{Y}$ represent the sample means of the relevant variables. An r value closer to +1 indicates a strong positive linear relationship, a value closer to −1 indicates a strong negative linear relationship, and values closer to 0 indicate a weak linear relationship. The statistical significance of the Pearson correlation coefficient was evaluated using a two-tailed *t*-test, and the test statistic was calculated using Equation (14).

(14) $$t=\frac{r\times \sqrt{n-2}}{\sqrt{1-r^{2}}}\;\;df=n-2$$

Here, *n* represents the number of observations, and df represents the degrees of freedom. The obtained *p*-values were used to evaluate whether the correlation coefficient was significantly different from zero. However, due to the time series structure and the possibility of a common long-term trend, Pearson correlations were interpreted not as an indicator of causal effect, but as an indicator of linear covariation between temperature anomaly and beekeeping indicators.

#### 2.4.2. Spearman Rank Correlation

It was calculated as a rank-based alternative to the Pearson coefficient, independent of the linearity assumption, and expressed by Equation (15).

(15) $$\rho =1-\frac{6\times \sum d_{i}^{2}}{n\left(n^{2}-1\right)}$$

Here, d_i_ represents the rank difference of each pair of observations. Similar values of the Pearson and Spearman coefficients indicate that replacing the original observations with their ranks did not substantially change the estimated strength or direction of the association; this does not by itself confirm that the underlying relationship is both linear and monotonic, since both coefficients can be similarly elevated by a shared long-term trend even if year-to-year changes are not closely related (see Section 2.4.4).

#### 2.4.3. Bootstrap Confidence Interval

In the bootstrap procedure, the number of resamples was set as B = 2000, and the random number generator was initialized with a fixed seed value (seed = 42) to ensure the reproducibility of the results. A moving-block bootstrap was used instead of observation-wise resampling, to preserve short-range temporal dependence. Consecutive observations were grouped into blocks of length ℓ = ⌈0.15 × *n*⌉ years; in each of the B = 2000 iterations, ⌈*n*/ℓ⌉ blocks were resampled with replacement and concatenated (then truncated to length *n*), and the Pearson r coefficient was recalculated on the resulting block-resampled series. The 95% confidence interval was calculated using Equation (16) with the 2.5th and 97.5th percentile values of the bootstrap distribution.

(16) $$95\%\;CI=\left[\mathrm{percentile}\left(r_{boot},2.5\right),\;\mathrm{percentile}\left(r_{boot},97.5\right)\right]$$

The use of a fixed seed (seed = 42) ensures the reproducibility of the results. A moving-block bootstrap (block length ≈ 15% of *n*) was therefore adopted in place of observation-wise resampling to better preserve short-range temporal dependence between consecutive years, although it does not fully replicate the dependence structure of an explicit time-series model. Accordingly, bootstrap confidence intervals have been considered as a complementary reliability indicator of the sampling stability of the correlation coefficients; they have not been interpreted as a method that eliminates time series dependence.

#### 2.4.4. Interpretation of Time Trends and Correlations

Each panel reports Pearson r, bootstrap 95% confidence interval, and Spearman ρ values together. The temperature anomaly, colony number, and honey production value series contain significant long-term trends during the 1961–2024 period. Therefore, Pearson and Spearman correlation coefficients calculated from the level series may be affected by the common temporal trend rather than the direct short-term relationship between the variables. In this study, correlation analyses on the level series were evaluated as descriptive long-term relationship indicators. To reduce the risk of misleading correlations that may arise from common trends, cross-correlation function (CCF) analysis was additionally applied to the first-differenced series. Therefore, the correlation results were interpreted not as causal effects, but as statistical covariation between temperature anomaly and beekeeping indicators.

### 2.5. Country-Based OLS Regression Analysis

Two separate OLS models were established for each Mediterranean country; the colony number model is defined by Equation (17), and the honey production value model is defined by Equation (18):

(17) $$\mathrm{Model}\;1:\;Colony_{i}\left(t\right)=\alpha_{i}+\beta_{i}\times Temp_{i}\left(t\right)+\varepsilon_{i}\left(t\right)$$

(18) $$\mathrm{Model}\;2:\;Honey_{i}\left(t\right)=\alpha_{i}+\beta_{i}\times Temp_{i}\left(t\right)+\varepsilon_{i}\left(t\right)$$

Annual observations from consecutive years are not independent, and classical OLS standard errors were therefore not used. Standard errors were instead calculated with the Newey-West heteroskedasticity and autocorrelation consistent (HAC) estimator given in Equation (19). The truncation lag was selected by the Bartlett rule as L = ⌊4(*n*/100)^(2/9)^⌋, and Bartlett kernel weights w(v) = 1 − |v|/(L + 1) were applied. The regression coefficients are the same under both estimators; only the standard errors, confidence intervals and *p* values change. Standardized coefficients were also calculated by regressing the z-scored response variable on the z-scored temperature anomaly, where z(a) = (a − ā)/s_a, so that countries with different sector sizes could be compared.

(19) $${\mathrm{Var}}_{\mathrm{HAC}}\left(\hat{\beta }\right)={\left(X^{\prime }X\right)}^{-1}\left[\sum_{v=-L}^{L}w\left(v\right)\;\Gamma \left(v\right)\right]{\left(X^{\prime }X\right)}^{-1}$$

Since the model was estimated separately for each country, the constant α_i partially represents the country’s own initial level and country-specific mean differences that remain constant over time. However, this approach is not a combined panel fixed effects model in the classical sense; therefore, the analysis was interpreted as country-specific discrete time series OLS regressions. This method was used to comparatively examine the within-country linear relationships between temperature anomaly and colony number and honey production value. Interpretation of the β coefficient: Change in average colony number (units) or change in honey production value (constant price Int$) associated with a 1 °C temperature increase. Significance levels were reported as follows: *p* < 0.05, *p* < 0.01, *p* < 0.001. The minimum observation threshold was increased to *n* ≥ 20 country-years; Montenegro (*n* = 19) was therefore excluded from all country-specific regressions (Supplementary Table S4a,b). Standard errors were estimated using Newey–West (HAC) heteroskedasticity- and autocorrelation-consistent standard errors, with the truncation lag selected via the Bartlett rule: lag = max(1, ⌊4·(*n*/100)^(2/9)⌋). Regression coefficients (β) are numerically identical between classical OLS and HAC estimation; only standard errors, confidence intervals, and *p*-values differ. In addition, standardized coefficients (temperature and response variable both z-scored prior to regression) are reported in Supplementary Table S4a,b to allow cross-country comparison independent of the absolute scale of each country’s beekeeping sector. Therefore, *p*-values were evaluated for descriptive comparison purposes; they were not interpreted as definitive evidence of significance completely free from time series autocorrelation.

### 2.6. Cross-Correlation Function—CCF

Cross-Correlation Function (CCF) was used to evaluate lagged relationships between temperature anomalies and beekeeping indicators. The maximum lag was set at 10 years to allow detection of both short-term (same-year to few-year) associations, which are considered more biologically and economically plausible for honey production value, and longer-term structural associations, which may be more relevant for registered colony numbers given that colony-number changes partly reflect slower-moving sectoral capacity expansion. Because the number of overlapping observation pairs decreases and the number of lag-specific comparisons increases with the maximum lag, a robustness check was additionally performed using a shorter maximum lag of 5 years; the pattern of lag-specific associations under this shorter range is reported in Section 3.5. The analysis was applied both on the original level series and on the first-differenced series to reduce the effect of common long-term trends.

#### 2.6.1. CCF on Original Series

The series was first converted to z-scores using Equations (20) and (21), then Pearson correlation was calculated for each lag value using Equation (22):

(20) $$X_{norm}=\frac{X-\mu_{X}}{\sigma_{X}}$$

(21) $$Y_{norm}=\frac{Y-\mu_{Y}}{\sigma_{Y}}$$

(22) $$CCF\left(lag=l\right)=\mathrm{cor}\left(X_{norm}\left(1:n-l\right),\;Y_{norm}\left(l+1:n\right)\right)\;\;l=0,1,\ldots ,10$$

Here, X_norm(t) represents the z-score of the temperature anomaly series; Y_norm(t) represents the z-score of the colony number or honey production value series; μ and σ represent the mean and standard deviation of the respective series, respectively; and l represents the lag length. A positive lag l indicates that the beekeeping indicator (colony number or honey production value) in year t + l is compared against the temperature anomaly in year t; that is, temperature leads the beekeeping indicator by l years.

#### 2.6.2. CCF on Differenced Series (Trend Correction)

Since both series contain long-term trends, the CCF values calculated from the original series may be affected by the common temporal trend, which may lead to the relationships appearing stronger than they are. To reduce this problem, the series were first subjected to z-score normalization with Equation (23), and then the first difference of the z-scored series was taken with Equation (24). The cross-correlation coefficients on the differenced series were calculated using Equation (25) for each lag value.

(23) $$X_{norm}=\frac{X-\mu_{X}}{\sigma_{X}}$$

(24) $$\Delta X_{norm}\left(t\right)=X_{norm}\left(t\right)-X_{norm}\left(t-1\right)\;\;t=2,\ldots ,n$$

(25) $$CCF_{diff}\left(lag=l\right)=\mathrm{cor}\left(\Delta X_{norm}\left(1:n-1-l\right),\;\Delta Y_{norm}\left(l+1:n-1\right)\right)$$

Here, X_norm represents the z-scored value of the relevant series; μ_X and σ_X represent the mean and standard deviation of the relevant series, respectively; and ΔX_norm(t) represents the first difference of the z-scored series. In the CCF_diff calculation, the X series represents the temperature anomaly, and the Y series represents the colony number or honey production value series. ΔY_norm was obtained by applying the same z-score normalization and first difference calculation to the Y series.

(26) $$\mathrm{Band}\left(l\right)=\pm \frac{1.96}{\sqrt{n-l}}\;\;l=0,1,\ldots ,10$$

The confidence band was calculated separately for each lag, since the number of overlapping observation pairs decreases from *n* at lag 0 to *n* − l at lag l (Equation (26)). This band represents an approximate pointwise 95% limit under near-independence assumptions and is not corrected for the fact that 11 lag-specific coefficients were examined per response variable; consequently, all CCF results are reported as exploratory rather than confirmatory.

### 2.7. Country-Based Regression of Honey Production Value per Colony

The linear relationship between the normalized honey production value per colony index and the temperature anomaly for each country was calculated using OLS with Newey–West (HAC) heteroskedasticity- and autocorrelation-consistent standard errors (see Section 2.5), and defined by Equation (27). In this analysis, the dependent variable is the constant price of honey production value per colony index normalized for each country’s own starting year. The independent variable is the annual temperature anomaly value of the same country.

(27) $$EffNorm_{i}\left(t\right)=\alpha_{i}+\beta_{i}\times Temp_{i}\left(t\right)+\varepsilon_{i}\left(t\right)$$

Here, EffNorm_i(t) represents the normalized honey production value per colony index for country i in year t; Temp_i(t) represents the temperature anomaly value for the same country in year t; α_i is the country-specific constant term; β_i is the regression slope (not a correlation coefficient) relating temperature anomaly to normalized honey production value per colony index; and ε_i(t) is the error term. A β_i > 0 indicates a positive linear relationship between the temperature anomaly and the normalized honey production value per colony index; a β_i < 0 indicates a negative linear relationship. These coefficients were interpreted not as direct biological changes in physical honey yield, but as the relationship between the temperature anomaly and a normalized index calculated based on the economic production value at a constant price. Because the magnitude of β_i depends on the single-year (t_0_) normalization baseline, a sensitivity analysis was conducted using a 3-year (t_0_, t_0_ + 1, t_0_ + 2) baseline mean instead of the single starting year; the results of this sensitivity analysis are reported in Section 3.6.

### 2.8. Software and Reproducibility

All analyses were performed using Python 3.13.5 (Python Software Foundation, Wilmington, DE, USA; https://www.python.org) with the Anaconda distribution (Anaconda Inc., Austin, TX, USA; https://www.anaconda.com). The library versions used were directly verified from the Python environment where the analyses were run and are as follows: pandas 3.0.2, numpy 2.1.3, matplotlib 3.10.8, seaborn 0.13.2, scipy 1.17.1 and statsmodels 0.14.6. To ensure the reproducibility of the resampling process in the bootstrap analysis, a fixed seed value was used for the random number generator (random seed = 42). The analysis workflow is reproducible when using the same FAOSTAT data version and the same code. The analysis scripts and processed datasets are available at Zenodo (https://doi.org/10.5281/zenodo.21861135, accessed on 9 August 2026). All images were saved in PNG format with a resolution of 300 DPI.

### 2.9. Stationarity and Autocorrelation Diagnostics

**Figure 1:**
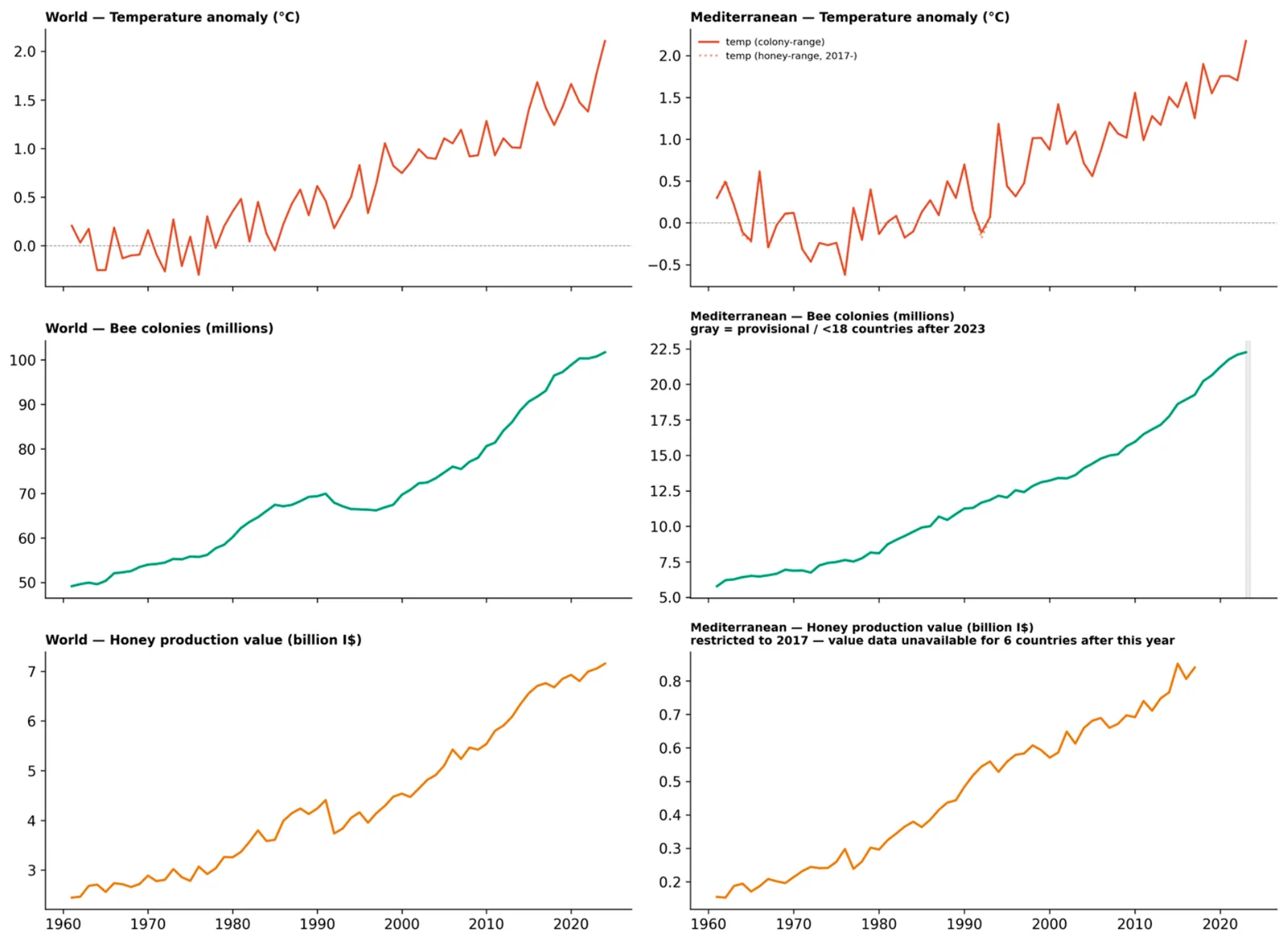
Temperature anomaly, registered colony number, and constant-price honey production value, World and Mediterranean basin, 1961–2024, shown in separate (non-dual-axis) panels. Mediterranean colony series extend to 2023 with complete 18-country coverage; honey production value series are restricted to 1961–2017 for the same reason. Shaded regions indicate years with reduced country coverage. Temperature anomaly is expressed in °C relative to the FAOSTAT 1951–1980 baseline; colony number is shown in millions of colonies and honey production value in billions of constant-price international dollars.

To formally assess whether the observed level-series correlations may be driven by common long-term trends rather than short-term co-movement, the stationarity properties of all analysed series were examined using the Augmented Dickey–Fuller (ADF) test (null hypothesis: the series contains a unit root/is non-stationary) and the KPSS test (null hypothesis: the series is stationary). Both tests were applied to the original level series (temperature anomaly, colony number, honey production value, and the normalized efficiency index) at the World and Mediterranean scales, and to the corresponding first-differenced series. In addition, the Ljung–Box test was applied to the first-differenced series to assess whether residual serial correlation remained after differencing. All level series were found to be non-stationary, consistent with the pronounced long-term trends visible in Figure 1. Most first-differenced series were stationary; however, residual autocorrelation remained in the first-differenced colony and honey production value series (Ljung–Box *p* = 0.0025 and *p* = 0.032, respectively, at lag 10), indicating that first differencing reduces but does not fully remove serial dependence. This result directly motivates the exploratory (rather than confirmatory) interpretation of the lag-specific cross-correlation findings reported in Section 3.5. Full test statistics for all series are reported in Supplementary Table S1.

### 2.10. Leave-One-Country-Out Sensitivity Analysis

Because Türkiye accounts for a disproportionately large share of Mediterranean colony numbers and honey production value in absolute terms, a leave-one-country-out sensitivity analysis was conducted to assess whether basin-level associations are driven predominantly by this single country. The basin-level temperature–colony number correlation was recalculated after excluding Türkiye from the Mediterranean panel, using the same aggregation procedure described in Section 2.2. The correlation changed only marginally when Türkiye was excluded (r = 0.896 with Türkiye included versus r = 0.892 excluded; Δr = 0.004), indicating that while Türkiye contributes substantially to the absolute scale of Mediterranean colony numbers and honey production value (Section 3.1; Supplementary Table S3), it is not the primary driver of the basin-level temperature–colony correlation reported in Section 3.3. This check was performed for the level-series correlation only; corresponding leave-one-out analyses for the cross-correlation function and bootstrap interval width were not conducted and are noted as a limitation in Section 4. Full results are reported in Supplementary Table S3.

## 3. Results

### 3.1. Time Series Trend (1961–2024)

Time series trends for the world and the Mediterranean basin, are presented in two separate panels (Figure 1). Each variable is shown in a separate panel, with colony number and honey production value normalized to 1961 = 100. In the world panel, the temperature anomaly reached +2.11 °C in 2024 from +0.21 °C in 1961; the corresponding warming rate is approximately 0.29 ± 0.02 °C per decade (OLS trend, *p* < 0.001). The colony number index increased from 100 to approximately 205 in the same period, and the honey production value index increased from 100 to approximately 295; that is, the honey production value approximately tripled in six decades. The picture is even more striking in the Mediterranean panel: the temperature anomaly, calculated using the equally weighted average of 18 countries, reached +2.18 °C in 2023 (the last full-coverage year for the colony-based series), corresponding to a warming rate of approximately 0.33 ± 0.03 °C per decade and was approximately 0.41 °C above the world average. During the same period, the colony index reached approximately 385, and the honey production value index reached its maximum in 2015 at approximately 551. However, since this temperature value is not an area-weighted basin average, but an equally weighted average of the countries included in the analysis, the results should be interpreted as a country-based regional indicator. These figures indicate that the Mediterranean basin had a higher temperature anomaly value in 2023–2024 and a higher decadal warming rate (0.33 vs. 0.29 °C/decade) than the world average; however, because the Mediterranean series is an equally weighted country average rather than an area-weighted physical estimate, this comparison should be regarded as descriptive rather than as evidence of a stronger climatic effect. However, the increase in the number of colonies should not be considered a direct biological consequence of the temperature increase; this increase also reflects the expansion of the institutional, economic, and technical capacity of the beekeeping sector. Türkiye plays a major role in the basin’s colony and honey-value growth (Supplementary Table S3); however, a leave-one-country-out sensitivity analysis shows that excluding Türkiye changes the basin-level temperature–colony correlation only marginally (r = 0.896 with Türkiye vs. r = 0.892 without; Δr = 0.004), indicating that Türkiye’s influence on this particular correlation, while substantial in absolute contribution, is not decisive for the overall association. According to FAOSTAT data, the number of registered colonies in Türkiye was approximately 1.49 million in 1961, while it approached approximately 9 million by 2023. Türkiye’s share of colonies within the Mediterranean basin rose from approximately 26% in 1961 to approximately 41% in 2023; in terms of honey production value, its share rose from approximately 19% in 1961 to approximately 49% in 2017. The apparent decline in the Mediterranean colony series after 2023 and in the honey-value series after 2017 reflects the reduction in reporting countries from 18 to 12 (Croatia, France, Greece, Italy, Slovenia, and Spain; Supplementary Table S2b), not a biological or sectoral contraction. Endpoint comparisons and trend summaries in this study are therefore restricted to the last year in which all 18 countries reported for each variable (2023 for colony number, 2017 for honey production value).

### 3.2. Sensitivity Analysis for the Mediterranean Temperature Series

**Figure 2:**
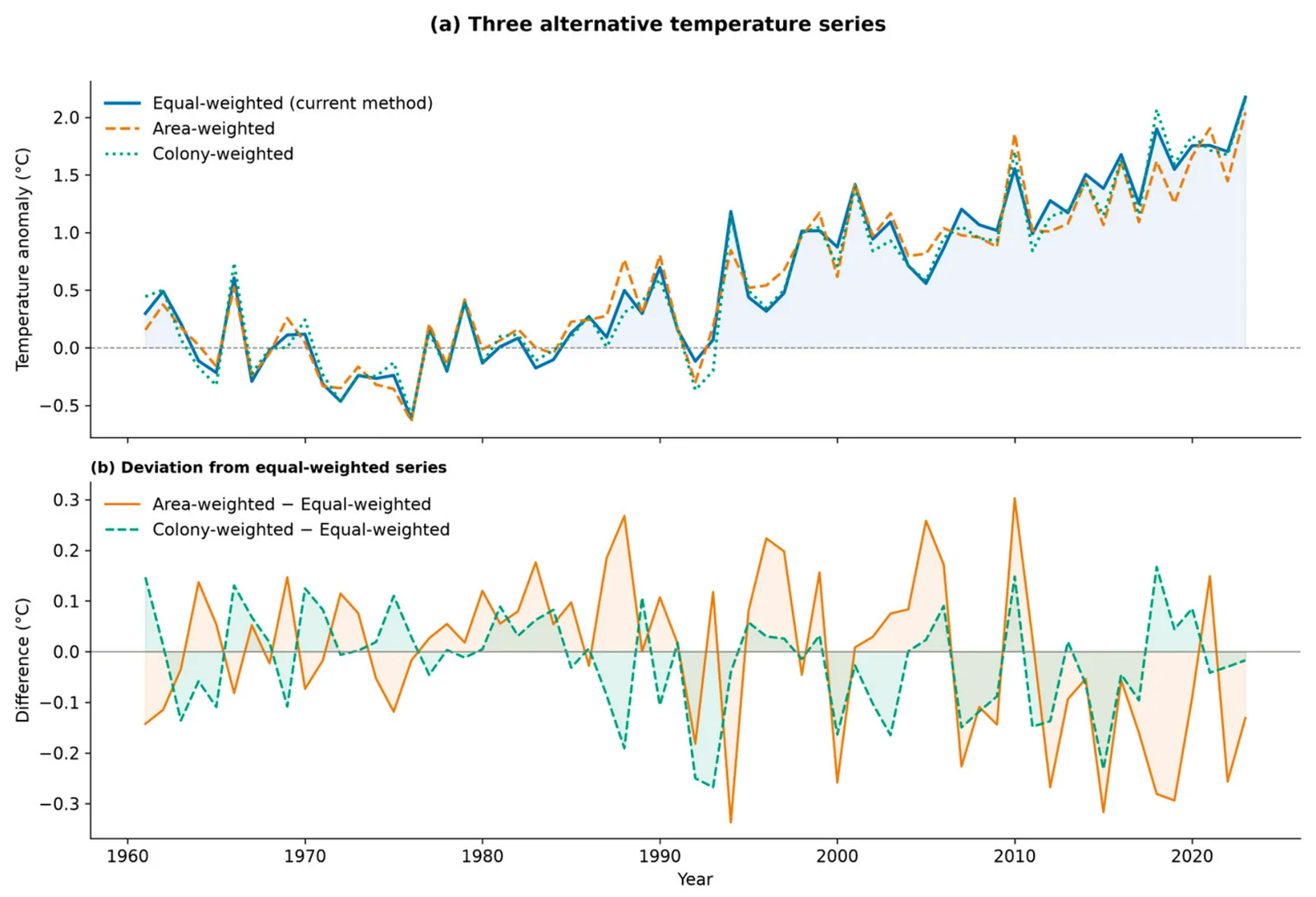
Comparison of equal-weighted, area-weighted, and colony-weighted Mediterranean temperature anomaly series.

In the main analysis, the Mediterranean temperature anomaly was calculated as the equally weighted arithmetic mean of the 18 countries included. To evaluate the possible impact of this approach on the results, the equally weighted temperature series was compared with the area-weighted temperature series according to country surface area and the colony-weighted temperature series according to annual colony count. The equally weighted, area-weighted, and colony-weighted temperature anomaly series showed similar long-term trends during the 1961–2023 period; the annual deviations of the area-weighted and colony-weighted series from the equally weighted series remained limited (Figure 2). The largest absolute deviations from the equally weighted series were 0.34 °C for the area-weighted series (in 1994) and 0.27 °C for the colony-weighted series (in 1993). A high agreement was found between the equally weighted temperature series and the area-weighted series (r = 0.9765; RMSE = 0.1515 °C; MAE = 0.1227 °C), and an even higher agreement was found between the equally weighted series and the colony-weighted series (r = 0.9896; RMSE = 0.1014 °C; MAE = 0.0788 °C). This finding indicates that the three temperature indicators are highly correlated in their level series; however, because all three share the same long-term warming trend, this level-series agreement does not by itself demonstrate robustness of the substantive temperature–beekeeping relationship (see first-differenced results, Section 3.3).

**Figure 3:**
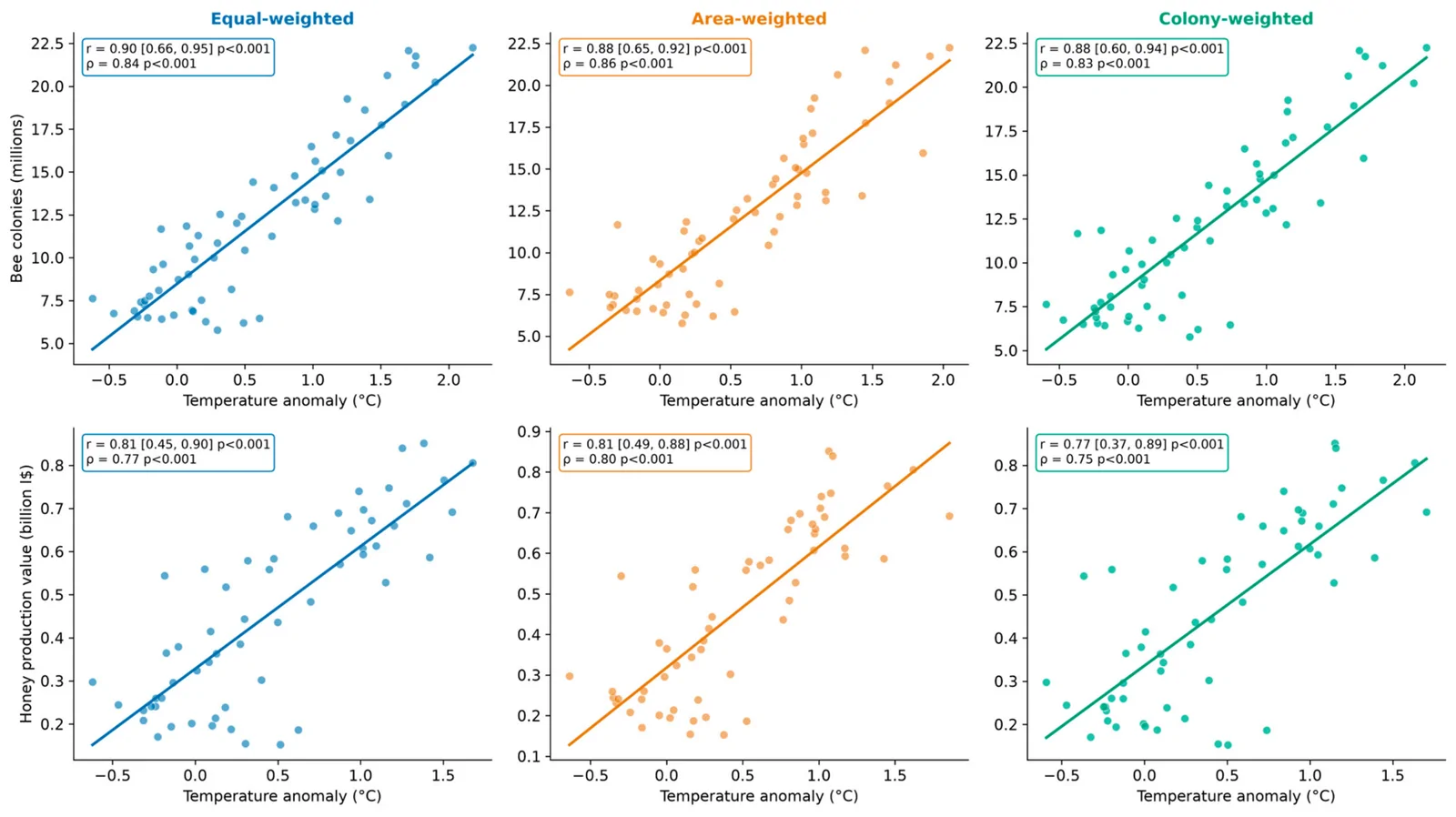
Correlation results according to alternative temperature series in the Mediterranean basin. Colony panels: 1961–2023 (18/18 countries). Honey panels: 1961–2017 (18/18 countries).

When alternative temperature series were used, the general direction of the correlation coefficients was preserved (Figure 3). In the Mediterranean basin, the temperature–colony relationship was calculated as r = 0.90 in the equally weighted series, r = 0.88 in the area-weighted series, and r = 0.88 in the colony-weighted series. The temperature-honey production value relationship was found to be r = 0.81, r = 0.81, and r = 0.77, respectively. The fact that the relationships remained positive in all alternative temperature indicators shows that the correlation findings are generally stable against the weighting of the temperature series. Additionally, basin-level OLS coefficients did not change directionally among alternative temperature series. Colony number was expressed in millions of colonies and honey production value in millions of constant-price international dollars (Int$) prior to regression; under this scaling, β coefficients for colony number remained in the range of 6.040–6.437 (million colonies per 1 °C), and for honey production value in the range of 281.206–297.824 (million I$ per 1 °C). All basin-level and country-level regressions in this study use Newey–West (HAC) standard errors (Section 2.5). These results demonstrate that the descriptive level-series correlations are similar across the three weighting schemes; robustness to the time-series assumptions underlying the analysis (non-stationarity, autocorrelation) is addressed separately in Section 3.3 via first-differenced correlations.

### 3.3. Correlation Results

**Figure 4:**
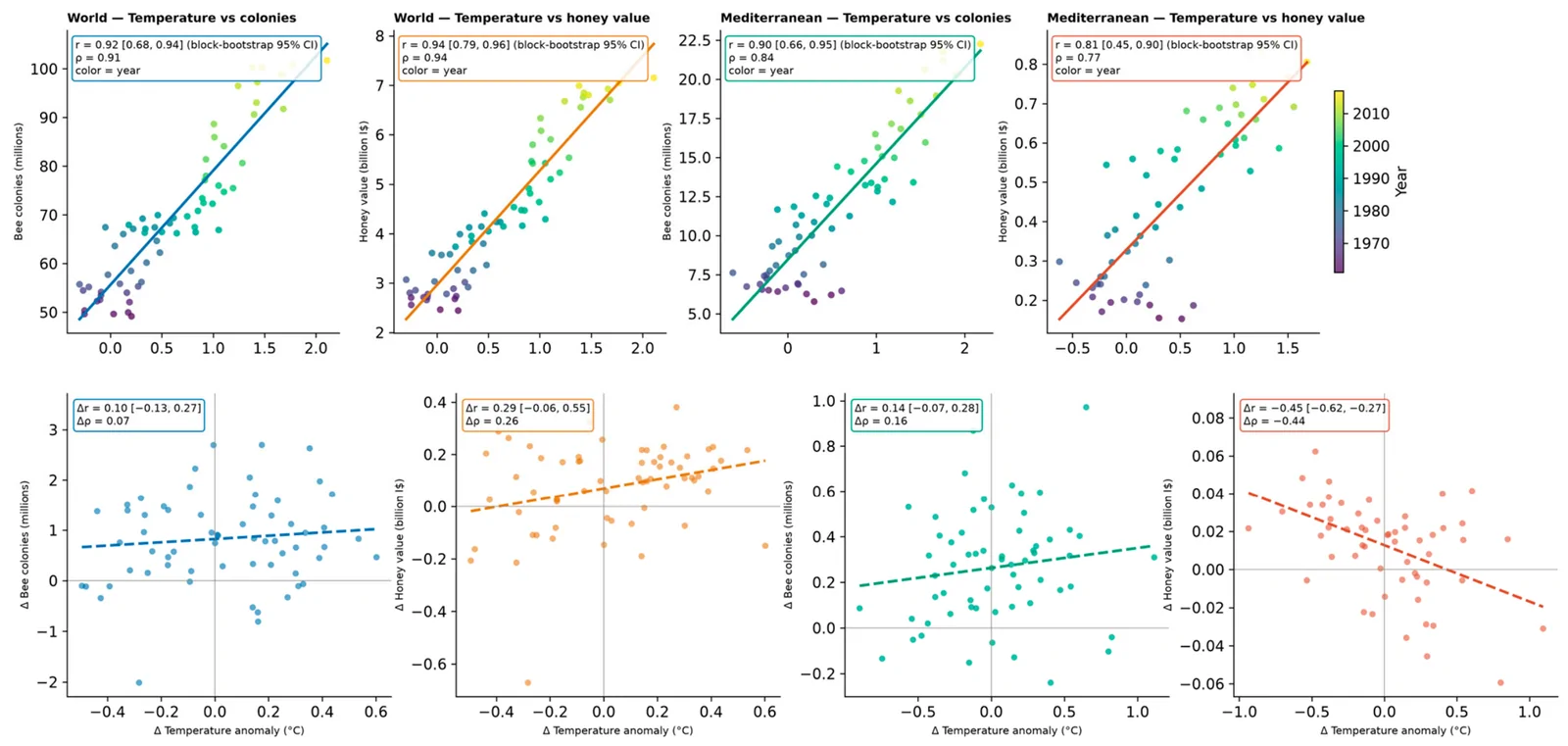
Correlation between temperature anomaly and registered colony number/constant-price honey production value. (**Top row**): level series, shown for description only; points are colored by year, and the apparent association largely follows the chronological progression of the series. (**Bottom row**): first-differenced (year-to-year change) series with moving-block bootstrap 95% confidence intervals. The panels are projections of two time series against each other and should not be read as independent cross-sectional observations.

The main correlations between temperature anomaly and colony number and honey production value are shown with four separate scatter plots for the world and the Mediterranean basin (Figure 4). Pearson r, bootstrap 95% confidence interval and Spearman ρ values are reported together in each panel. However, since these correlations were calculated from level series, the values may have been affected by a common long-term increasing trend. Therefore, the results in Figure 4 should be interpreted not as causal or short-term effect indicators, but as descriptive long-term covariation indicators for the period 1961–2023 (colony number) and 1961–2017 (honey production value). Globally, a correlation of r = 0.92 [0.68, 0.94] and ρ = 0.91 was found between temperature and colony number, and r = 0.94 [0.79, 0.96] and ρ = 0.94 between temperature and honey production value; both relationships are strong at the *p* < 0.001 level. These strong positive level-series correlations reflect long-term co-movement rather than a direct effect of temperature on colony number or honey production value. First-differenced (year-to-year) correlations are markedly weaker and, in one case, reverse in sign: World temp–colony Δr = 0.10 [−0.13, 0.27], World temp–honey Δr = 0.29 [−0.06, 0.55], Mediterranean temp–colony Δr = 0.14 [−0.07, 0.28], and Mediterranean temp–honey Δr = −0.45 [−0.62, −0.27] (Figure 4, bottom row; Supplementary Table S5). This divergence indicates that the strong level-series associations are driven substantially by shared long-term trends rather than by consistent short-term covariation, and is especially pronounced for Mediterranean honey production value. The number of colonies worldwide increased from approximately 49 million to 102 million in a temperature range extending from −0.30 °C to +2.11 °C. In the Mediterranean basin, the number of colonies increased from approximately 6 million to 22 million between −0.62 °C and +2.18 °C, with r = 0.90 [0.66, 0.95] and ρ = 0.84. A significant portion of this growth originated from Türkiye; Türkiye alone accounted for approximately 47% of the basin’s colony increase. The relationship between honey production value and the Mediterranean is r = 0.81 [0.45, 0.90] and ρ = 0.77, a numerically lower level-series correlation than the world average (r = 0.94); part of this difference may reflect intra-basin country heterogeneity, though this explanation is not formally tested here beyond the leave-one-out check in Supplementary Table S3. Therefore, correlation coefficients should not be interpreted as directly indicating that temperature increase increases colony numbers or honey production value; rather, they should be evaluated in conjunction with the long-term growth of the beekeeping sector, national scale, production capacity, and changes in record-keeping systems.

### 3.4. Country-Based OLS Regression Results

**Figure 5:**
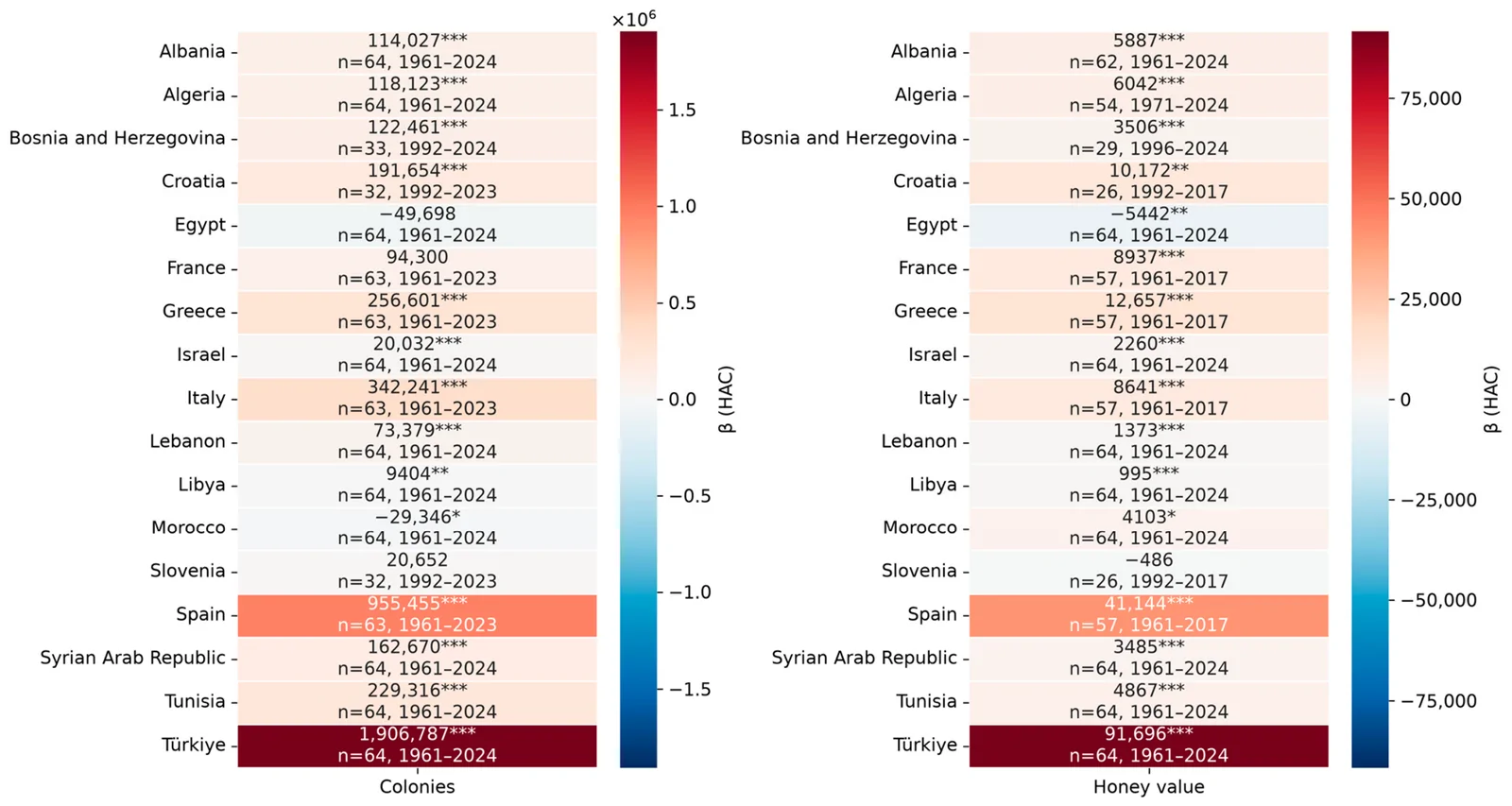
Country-specific regression slopes (β) relating temperature anomaly to registered colony number and to constant-price honey production value, estimated with Newey–West (HAC) standard errors (minimum *n* = 20 country-years). Separate diverging color scales centered at zero are used for the two outcomes, which are expressed in different units and are not directly comparable. Cell labels show the coefficient, the number of observations, and the observation period. Coefficients are reported in original units and should not be compared across countries; standardized coefficients are given in Supplementary Table S4a,b. Asterisks denote statistical significance based on HAC standard errors: *** *p* < 0.001, ** *p* < 0.01, * *p* < 0.05.

Country-based OLS regression coefficients are summarized in a heat map format for 18 Mediterranean countries (Figure 5). The statistical significance level of the corresponding coefficient in each cell is indicated by stars. Significance levels are based on Newey-West (HAC) standard errors, which are robust to heteroskedasticity and serial correlation; sample sizes, observation periods, standardized coefficients and residual diagnostics for each country are reported in Supplementary Table S4a,b. The largest coefficient for colony number is observed for Türkiye (β = 1,906,787; HAC *p* < 0.001; standardized β = 0.72), indicating that years with a temperature anomaly 1 °C higher were associated with approximately 1.9 million more registered colonies in this level-series regression. Because the magnitude of this raw coefficient depends on the absolute scale of Türkiye’s beekeeping sector, it should not be read as indicating the strongest relationship in the basin; standardized coefficients allowing scale-independent comparison are given in Supplementary Table S4a. This coefficient should not be interpreted as a causal biological increase, but rather as a statistical reflection of the strong expansion of beekeeping capacity in Türkiye during the same period, along with the temperature anomaly. Spain (β = 955,455; HAC *p* < 0.001), Italy (β = 342,241; HAC *p* < 0.001), Greece (β = 256,601; HAC *p* < 0.001) and Tunisia (β = 229,316; HAC *p* < 0.001) are other countries with statistically significant positive coefficients relating temperature anomaly to the number of registered colonies. In contrast, Morocco shows a significant negative coefficient (β = −29,346; HAC *p* = 0.011), while the coefficients for France (β = 94,300; HAC *p* = 0.226) and Egypt (β = −49,698; HAC *p* = 0.448) are not statistically significant once autocorrelation-robust standard errors are used. For honey production value, the largest coefficients are again observed for Türkiye (β = 91,696; HAC *p* < 0.001) and Spain (β = 41,144; HAC *p* < 0.001), followed by Greece (β = 12,657; HAC *p* < 0.001) and Croatia (β = 10,172; HAC *p* = 0.009). Morocco shows a negative coefficient for colony number (β = −29,346) alongside a positive coefficient for honey production value (β = 4103; HAC *p* = 0.027). This pattern is consistent with, but does not by itself demonstrate, higher production value per remaining colony; direct analysis of the honey-value-per-colony indicator (Section 3.6) would be required to support such an interpretation. Egypt is the only country in the basin with a clearly negative coefficient for honey production value (β = −5442; HAC *p* = 0.002). The consistently positive trends observed in Türkiye for both colony numbers and honey production indicate its important role in the Mediterranean beekeeping sector.

Methodologically, these results were obtained from country-specific time-series regressions with Newey-West (HAC) standard errors, not from a combined panel fixed-effects model. Therefore, the coefficients should be interpreted not as direct causal effect magnitudes between countries, but as the direction and relative magnitude of the intra-country linear relationship between beekeeping indicators related to temperature anomalies.

### 3.5. Cross-Correlation Function (CCF)

**Figure 6:**
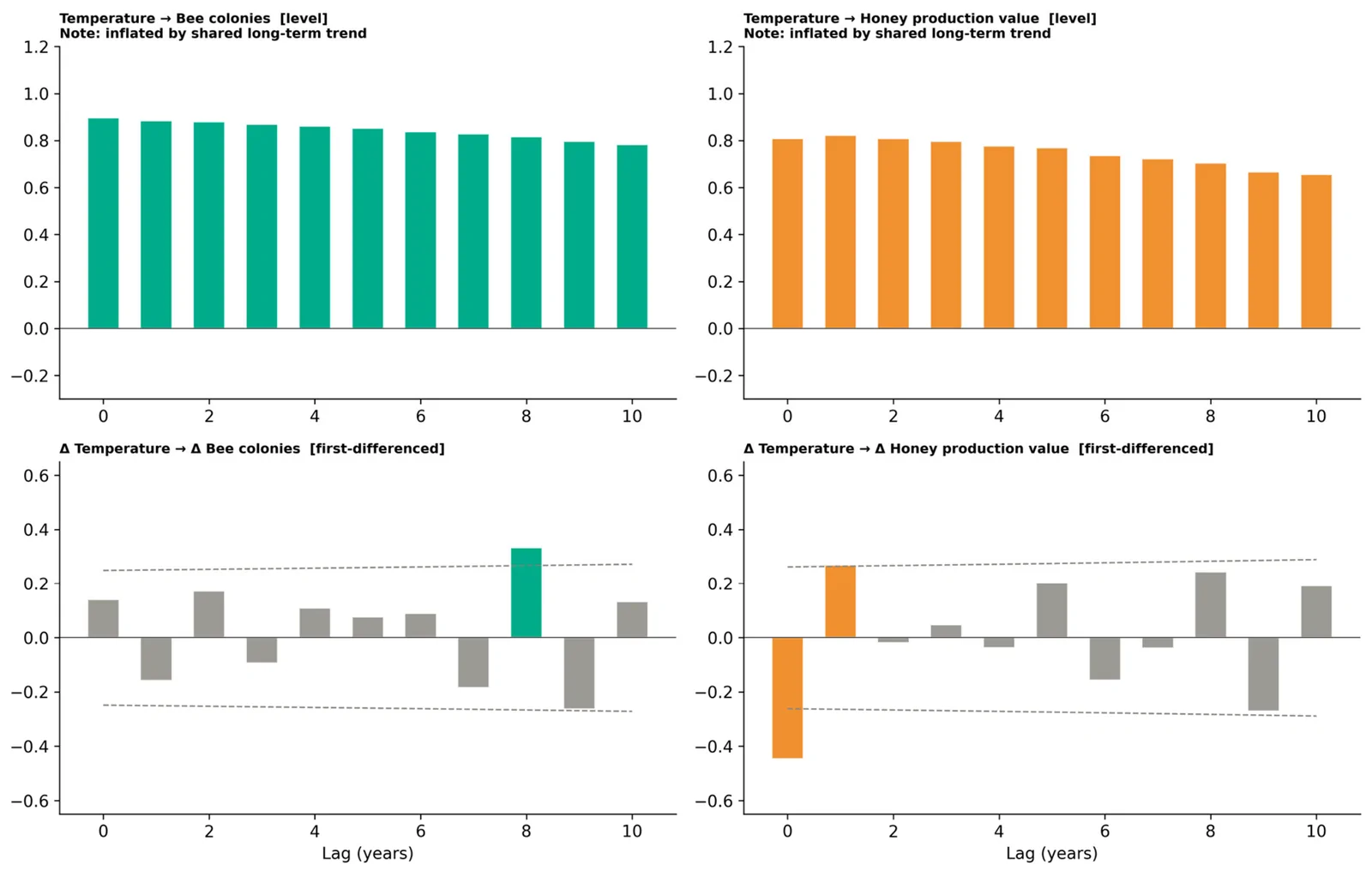
Cross-correlation function (CCF) between temperature anomaly and registered colony number/constant-price honey production value. A positive lag l denotes corr(temperature at year t, indicator at year t + l); that is, temperature leads the indicator by l years. The arrow in each panel title denotes the direction of the lag structure tested, not a causal relationship. (**Top row**): level series, shown for description only, as the coefficients are inflated by the shared long-term trend. (**Bottom row**): first-differenced series with approximate pointwise 95% bands calculated as ±1.96/√(*n* − l), which widen with lag as the number of overlapping observation pairs decreases. The bands are not corrected for the examination of 11 lag-specific coefficients per outcome, and the lag-specific findings are therefore exploratory. In the bottom row, colored bars mark lags exceeding the approximate pointwise 95% band and grey bars those that do not.

The cross-correlation results for the original and first-differenced series are compared in Figure 6. In the level series (Figure 6, top row), the temperature–colony correlation ranged from r = 0.90 at lag 0 to r = 0.78 at lag 10, and the temperature–honey production value correlation ranged from r = 0.81 at lag 0 to r = 0.65 at lag 10. Both panels are flagged as likely inflated by the shared long-term warming trend. In the first-differenced series (Figure 6, bottom row), the colony series exceeded the approximate pointwise 95% threshold only at lag 8 (r = 0.33). Countries with large colony stocks, such as Türkiye and Spain, plausibly contribute disproportionately to the basin-level CCF, though this was not formally tested via a country-level influence analysis beyond the single leave-one-out check reported for the level correlation (Supplementary Table S3). The honey series exceeded the threshold at lag 0 (r = −0.44) and lag 1 (r = 0.27). The lag-0 coefficient indicates contemporaneous covariation between year-to-year changes in temperature anomaly and year-to-year changes in constant-price honey production value. Because residual autocorrelation remained after differencing (Ljung–Box *p* = 0.0025 for colony and *p* = 0.032 for honey at lag 10; Supplementary Table S1), and because 11 lag-specific coefficients were examined per outcome without multiplicity correction, these first-differenced findings are reported as exploratory. Under the shorter 5-year lag range used as a robustness check, no lag exceeded the approximate pointwise threshold for the first-differenced colony series, consistent with the exploratory status of the lag-8 finding reported under the full 10-year range.

### 3.6. Honey Production Value Index per Colony

**Figure 7:**
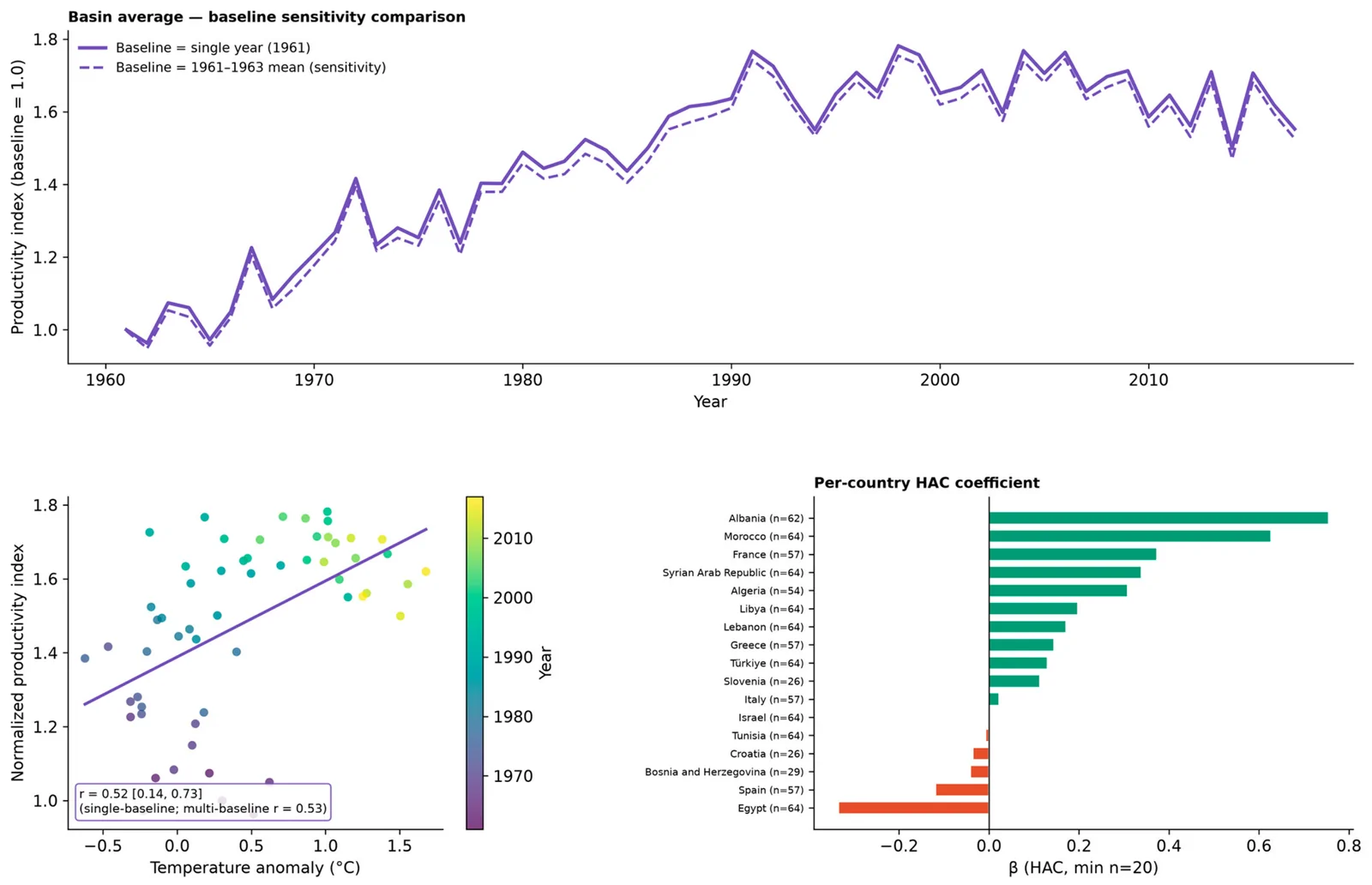
Normalized honey production value per colony index, Mediterranean basin. (**Top**): basin average over 1961–2017, the period with complete 18-country honey-value coverage, under single-year vs. 3-year baseline normalization. (**Bottom-left**): basin-average correlation with temperature anomaly over the same period. (**Bottom-right**): per-country regression slopes (β_i) estimated with Newey–West (HAC) standard errors over each country’s full reporting period (*n* ≥ 20; *n* shown per country). Slope magnitudes depend on each country’s normalization baseline and should not be read as indicating the strength of the relationship; standardized coefficients are given in Supplementary Table S4c.

The normalized honey production value index per colony provides a comparative overview of the change in economic production value per colony by reducing scale differences between countries (Figure 7). Each country is normalized to its initial value in its own data series, reducing scale differences between countries; this reduces, but does not eliminate, the dominance of countries with large absolute production values, and places the change in economic production value per colony for 18 countries on a comparable basis. The basin average, defined as 1.0 in the initial year (1961) in the upper panel, reached approximately 1.55–1.75 by 2017 (the last year with complete 18-country honey-value coverage), representing an increase of approximately 55–75% depending on whether a single-year or 3-year baseline is used (Figure 7, top panel). Within the gray lines, it is noteworthy that some countries have diverged substantially from the basin average, especially since the 1990s; this divergence should be read in conjunction with the country-based analysis section. In the lower left panel, r = 0.52 [0.14, 0.73] (single-year baseline) and r = 0.53 (3-year baseline sensitivity check) were obtained from the basin average, with block-bootstrap 95% confidence intervals. The color scale clearly reveals the temporal structure; a low production value index per colony in purple-blue tones is observed between 1960 and 1980, and a marked increase in yellow-green tones is seen towards the end of the series; the productivity index visually appears more pronounced in recent decades. However, no formal breakpoint or segmented-regression test was conducted to confirm statistically that this increase is temporally concentrated or that it coincides with an acceleration in warming, and this description should be read as a qualitative pattern rather than a tested result. In the country-based beta coefficients in the lower right panel, Albania (β ≈ 0.76) and Morocco (β ≈ 0.63) stand out as the countries showing the strongest positive relationship between the temperature anomaly and the normalized honey production value index per colony. Türkiye (standardized β ≈ 0.18, HAC *p* = 0.21, not statistically significant) ranks in the middle and is not statistically distinguishable from zero at conventional levels. Based on visual comparison of the separate colony-number and per-colony-value indicators, and not on a formal decomposition, Türkiye’s overall growth appears more related to the increase in the number of registered colonies and the total production value than to the increase in economic production value per colony. Egypt (β ≈ −0.33) and Spain (β ≈ −0.12) stand out as countries showing a negative relationship between the temperature anomaly and the normalized honey production value index per colony. This country-based divergence indicates that the relationship between the temperature anomaly and the honey production value index per colony is not homogeneous in the Mediterranean basin.

### 3.7. Bootstrap Confidence Intervals for Correlation Coefficients

**Figure 8:**
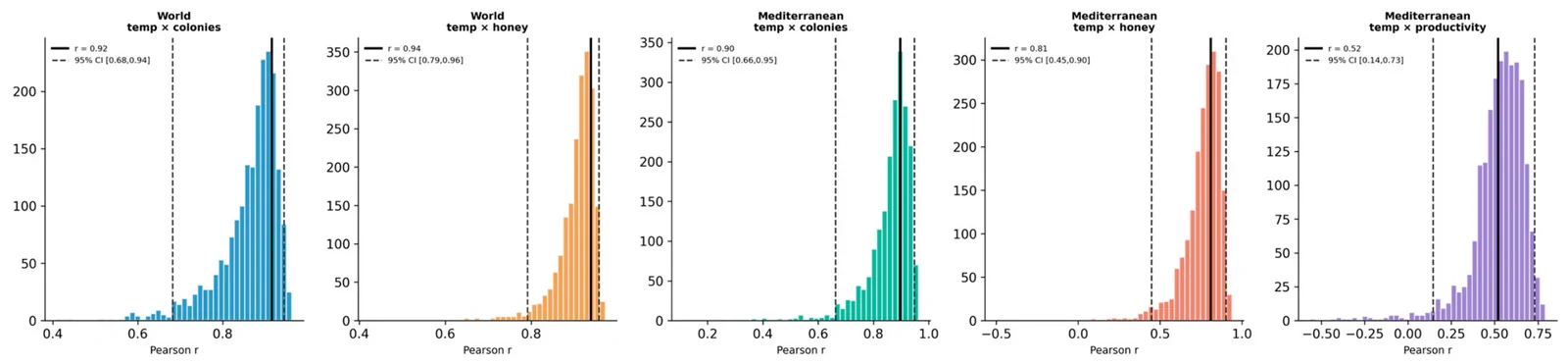
Moving-block bootstrap distributions of Pearson r for five temperature–indicator pairs. The resampling unit is the block of consecutive years (block length ≈ 15% of *n*), with 2000 replicates per pair; confidence intervals are percentile intervals (2.5th and 97.5th percentiles). Solid vertical lines mark the observed coefficient, dashed lines the bounds of the 95% interval.

The 2000-iteration bootstrap distributions of the five main correlation coefficients are presented in Figure 8. All distributions are clustered well away from zero under moving-block resampling, which preserves short-range temporal dependence better than observation-wise resampling. These results indicate that the correlation coefficients are stable under this block-resampling scheme; they do not, however, remove longer-range dependence or non-stationarity in the underlying level series. For the global temperature–colony relationship, r = 0.92 with a 95% CI of [0.68, 0.94]. For the global temperature–honey production value relationship, r = 0.94 with a 95% CI of [0.79, 0.96]. For the Mediterranean temperature–colony relationship, r = 0.90 with a 95% CI of [0.66, 0.95]; this width is consistent with greater within-basin heterogeneity relative to the global aggregate. For the Mediterranean temperature–honey production value relationship, r = 0.81 with a 95% CI of [0.45, 0.90]. For the normalized honey production value per colony index, r = 0.52 with a 95% CI of [0.14, 0.73] constitutes the widest band of the five. The lower bound remains above zero, but the interval is substantially wider than the others, and this coefficient should therefore be interpreted with greater caution.

## 4. Discussion

This study, using FAOSTAT data from 1961 to 2024, reveals that the relationship between temperature anomalies and recorded beekeeping indicators is not unidirectional, linear, or homogeneous; rather, it exhibits a complex structure that varies depending on scale, country, and the production indicator used. The findings demonstrate strong long-term positive relationships between temperature anomalies and the number of recorded bee colonies and the constant-price honey production value at both global and Mediterranean scales. However, these relationships should not be interpreted as causal evidence that temperature increase directly increases colony numbers or honey production. A more appropriate interpretation is that the beekeeping sector expanded during the same historical period when temperature anomalies increased, and that this expansion is a structural process shaped by economic, technological, managerial, and market-based factors. This indicates that the beekeeping sector is facing a multidimensional transformation process in the era of climate change, rather than a simple “collapse” or “benefit” dichotomy.

Sensitivity analyses regarding the method of calculating the temperature series also support this interpretation. The similar long-term trends of equally weighted, area-weighted, and colony-weighted Mediterranean temperature series, and the preservation of the direction of correlations under alternative temperature series, demonstrate that the main findings are largely stable against the choice of temperature indicator. Therefore, the findings should be considered not only as a result specific to the equally weighted country average, but also as a pattern of relationships that maintains its general direction in alternative weighting approaches. These findings are consistent with previous studies showing that managed honeybee colonies are increasing globally, but this increase is not occurring in parallel with the demand for agricultural pollination [6,11,12]. Global colony stocks are increasing, but this increase is lagging behind the demand for pollination-dependent agricultural production. Therefore, the increase in colony numbers does not mean that the fragility of beekeeping systems is decreasing. The positive long-term relationships observed in this study should also be considered as a synchronous movement showing that the sector is growing in conjunction with climate change. The increase in the number of colonies worldwide from approximately 49 million in 1961 to 102 million in 2024 reflects this sectoral expansion; however, it should not be overlooked that beekeeping technology, management practices, and global honey demand have also undergone radical transformations during the same period [6]. Studies conducted in southern Poland and the southern United Kingdom also observed an increase in honey yield and an extension of the production season with rising temperatures; however, they also showed that temperature altered harvest timing and production phenology. This suggests that the relationship between temperature anomalies and beekeeping indicators should be evaluated within a dynamic transformation process rather than a static cause-and-effect mechanism [11].

Cross-correlation analysis provides a critical methodological distinction in interpreting long-term relationships. A significant portion of the high correlations observed in the original time series stem from common long-term trends, and first-difference analyses allow for the separation of short-term covariation patterns from the common trend effect. A lagged association was found for colony number in the differenced series only at lag 8 (r = 0.33), against a background of residual autocorrelation that was not removed by first differencing; this result is treated as exploratory. This finding indicates that the increase in colony numbers is not a direct short-term response to annual temperature changes but rather is shaped by long-term structural dynamics such as beekeeping practices, management strategies, and the economic organization of the sector. Given that the number of registered colonies is affected by changes in beekeeping policies and official registration systems, this indicator should be interpreted as a sectoral capacity indicator rather than a measure of biological population dynamics. In contrast, honey production value shows a more pronounced covariation with short-term temperature anomaly changes. The negative correlation (r = −0.44) obtained at lag = 0 in the differential cross-correlation analysis indicates that sudden annual increases in temperature can suppress honey production value within the same year. This finding is biologically significant because short-term weather conditions can play a decisive role in honey production by affecting bee flight activity, nectar production, and nectar gathering behavior [19]. This short-term negative impact is consistent with findings from Mediterranean conditions where drought and high temperatures shorten the flowering period and reduce hive weight gain [8]. These results highlight that the time scale is a critical analytical parameter in evaluating the relationships between temperature anomalies and beekeeping indicators: while positive correlations are observed concurrently with sectoral growth in the long term, sudden temperature increases can put pressure on production value in the short term. The reversal in sign between the level and first-differenced series for honey production value is the most notable pattern in these results, and it illustrates why the two-time scales must be interpreted separately. Over six decades, both temperature anomaly and production value rose together, reflecting the parallel expansion of the sector during a period of sustained warming. Within individual years, however, warmer-than-usual conditions coincided with lower-than-usual production value. These two observations are not contradictory: the long-term association is dominated by structural sectoral change, whereas the year-to-year association is more likely to capture the sensitivity of production to seasonal conditions. This contrast also indicates that studies reporting only level-series correlations may substantially overstate the apparent benefit of warming for beekeeping output.

The country-specific variations observed in the Mediterranean basin more clearly highlight the context-sensitive nature of the relationship between temperature anomalies and beekeeping indicators. While positive correlations are observed between temperature anomalies and the number of registered colonies and honey production value in Türkiye, Spain, Tunisia, and Italy; these correlations are weak or negative in countries such as France, Morocco, and Egypt. This indicates heterogeneity in the direction and magnitude of the country-specific linear coefficients; this heterogeneity does not by itself establish non-linearity within countries or at the basin level, since non-linear models were not formally tested. Country-specific production structure, the status of floral resources, drought pressure, beekeeping practices, and adaptation capacity may play a role in this differentiation. This finding is consistent with the study highlighting that the effects of climate change on honeybees can vary depending on the context [12]. In a striking pattern, Morocco shows a negative coefficient on the colony side while exhibiting a positive correlation on the honey production value side; this asymmetry is consistent with, but does not demonstrate, higher economic value per remaining colony. Such an interpretation would require direct analysis of production value per colony together with information on physical yield and management inputs.

Given its dominant role in the Mediterranean basin, Türkiye’s basin share increased over the study period, reaching approximately 41% of total colony numbers and approximately 49% of honey production value by the end of the period analysed for each indicator (compared with approximately 26% and 19%, respectively, in 1961). The increase in the number of colonies from approximately 1.49 million in 1961 to nearly 9 million in 2023 represents more than a sixfold growth in six decades. However, this growth should be evaluated not only in terms of climatic conditions but also in conjunction with beekeeping policies, the spread of migratory beekeeping practices, and government support, although these mechanisms are plausible, literature-based explanations rather than variables examined directly in the present dataset. Therefore, the results obtained at the basin level reflect an average trend weighed towards production and do not mean that all countries responded in the same way.

One of the significant contributions of this study is its use of a normalized index of honey production value per colony, going beyond colony number and total production value. The findings show a general increase in the constant-price index of honey production value per colony in the Mediterranean basin over the long term, but this increase is not homogeneous among countries. Albania (β ≈ 0.76) and Morocco (β ≈ 0.63) exhibit the largest positive coefficients in the normalized index of honey production value per colony, while Türkiye ranks in the middle despite its absolute size and its coefficient is not statistically distinguishable from zero (standardized β ≈ 0.18; HAC *p* = 0.21). This finding suggests that sectoral growth in Türkiye is associated predominantly with growth in colony number, whereas in some smaller countries the increase is more evident in economic production value per colony; these descriptions refer to the observed indicators and are not based on a formal decomposition of production intensity. Egypt (β ≈ −0.33) stands out as the country showing the clearest negative coefficient in the normalized index of honey production value per colony. This situation reveals that the dynamics of total growth in the beekeeping sector and the economic production value per colony are distinct processes. These results should also be evaluated in the context of the effects of climate change on floral resources. Recent studies on a European scale show that increasing temperatures and decreasing rainfall can reduce the diversity of food sources utilized by honeybees and weaken the resilience of bee–plant interaction networks [10]. These findings point to a potential risk area for long-term production sustainability, particularly in climate-sensitive regions such as the Mediterranean. The long-term sustainability of beekeeping production in the Mediterranean basin may depend on the resilience of the floral resource base to climate change—a hypothesis grounded in the cited literature rather than directly tested in the present study, raising the possibility that a different relationship pattern may emerge in the future. Management strategies should be developed to mitigate the effects of climate change on honeybee colony losses [20]. Beekeepers should also be able to adopt strategies that can limit the negative impacts of climate change [21].

The analyses presented in this study could also provide a basis for a future predictive modelling framework. The distinction drawn here between long-term co-movement in the level series and year-to-year variation in the differenced series indicates that such a framework would need to represent at least two components: a slowly evolving structural component related to registered colony capacity and a shorter-term component associated with annual variation in production value. Such a model could estimate annual changes in recorded colony numbers and constant-price honey production value by combining temperature anomalies with precipitation, drought indices, land-use variables, disease pressure, and management- and policy-related indicators. Country-specific effects, unequal series lengths, non-stationarity and serial dependence would also need to be addressed explicitly. A panel time-series model, an autoregressive distributed lag specification, or a machine-learning approach with time-based validation could be evaluated for this purpose. The present dataset does not include the variables required for reliable prediction, and therefore the present findings are positioned as a basis for future model development rather than as a forecasting exercise.

Finally, the study has some limitations. Since the analysis is based on observational data, causality cannot be established. The dataset does not include variables such as precipitation, drought, extreme temperature events, land use, disease pressure, pesticide use, and beekeeping management. Although Newey–West (HAC) standard errors were used to address serial correlation, the level-series regressions remain descriptive and their coefficients should not be read as effect estimates. The leave-one-country-out check was performed for the level-series correlation only; corresponding checks for the cross-correlation function and for bootstrap interval width were not conducted. Residual autocorrelation remained after first differencing and prewhitening was not applied; the lag-specific cross-correlation results are therefore reported as exploratory, and their confirmation through prewhitening remains a task for future work with longer or higher-frequency series. Furthermore, the fact that the colony number variable reflects recorded beekeeping capacity rather than biological population dynamics creates limitations in the ecological interpretation of the findings. Overall, the results indicate that relationships between temperature anomalies and beekeeping indicators cannot be reduced to a simple pattern of increase or decrease. Instead, they are consistent with the combined operation of long-term structural change and shorter-term environmental variation, although the present analysis does not quantify or separate these processes.

## 5. Conclusions

This study shows that the long-term co-movement between temperature anomalies and recorded beekeeping indicators—registered colony numbers and constant-price honey production value—does not reflect a simple, causal, or homogeneous relationship. Level-series correlations are strong at both global and Mediterranean scales, but first-differenced analyses show this association weakens substantially and, for Mediterranean honey production value, reverses sign, suggesting that much of the level-series association reflects shared long-term trends rather than a consistent short-term relationship. The direction of the level-series associations was preserved under equally weighted, area-weighted and colony-weighted temperature indicators, indicating that the descriptive findings are not sensitive to the choice of weighting scheme. Registered colony numbers and the normalized honey production value per colony index behave as distinct sector-level indicators that do not always move together; this divergence, together with substantial heterogeneity across Mediterranean countries, indicates that the two indicators should be analysed and interpreted separately rather than treated as interchangeable measures of sectoral growth. These findings are restricted to the managed honeybee colonies and economic beekeeping indicators examined here and should not be generalized to pollinator systems more broadly.

Three recommendations follow from these findings. For research, colony number and economic production value per colony should be reported and modelled as separate indicators rather than combined into a single measure of sectoral performance, and long-term analyses should distinguish level-series co-movement from year-to-year variation before drawing conclusions about climatic sensitivity. For data infrastructure, the value of national reporting would be considerably increased if colony and production records were accompanied by information on registration criteria and management practices, since changes in these procedures are currently indistinguishable from changes in the sector itself. For future work, incorporating precipitation, drought indices, land-use change and management variables would allow the associations reported here to be examined within a framework capable of addressing mechanism rather than co-occurrence.

## Data Availability

The processed analytical datasets (Mediterranean panel and World series) and the analysis scripts supporting this study are openly available at Zenodo, https://doi.org/10.5281/zenodo.21861135 (accessed on 9 August 2026). The underlying raw data are publicly available from FAOSTAT; the domains, item and element codes, and extraction date are specified in Section 2.1.

## Supplementary Materials

The following supporting information can be downloaded at: https://www.mdpi.com/article/10.3390/insects17080867/s1, Table S1: stationarity and Ljung-Box autocorrelation tests for level and first-differenced series, Table S2: panel coverage by country and by year, Table S3: leave-one-country-out sensitivity for Türkiye, Table S4: full HAC panel regression results with standardized coefficients, sample sizes, and observation periods, Table S5: level-series and first-differenced correlations for all four temperature–indicator pairs, with block-bootstrap 95% confidence intervals and Spearman coefficients, and the processed analytical datasets (Mediterranean panel and World series) are available as Supplementary Files.

## Abbreviations

The following abbreviations are used in this manuscript:

CCF	Cross-correlation function
FAO	Food and Agriculture Organization of the United Nations
CI	Confidence interval
FAOSTAT	Food and Agriculture Organization Corporate Statistical Database
Int$	International dollars
MAE	Mean absolute error
OLS	Ordinary least squares
RMSE	Root mean square error
