# Supplementary material for: Temperature Anomalies and Structural Change in Global and Mediterranean Apiculture Systems
Source: Insects. 2026 Aug 20;17(8):867. doi: 10.3390/insects17080867 (PMC13513723; doi:10.3390/insects17080867)
Supplement: Supplementary file 1 [file insects-17-00867-s001.zip › insects-4445554-supplementary.pdf]

Supplementary Materials

Temperature Anomalies and Structural Change in Global and Mediterranean Apiculture Systems

Table S1. Stationarity and autocorrelation diagnostics

Augmented Dickey–Fuller (ADF; H0: unit root) and KPSS (H0: stationarity) tests, and Ljung–Box tests at lag 10, for each series in level form and in first-differenced form (prefix Δ). Series marked "colony-range" cover 1961–2023 and those marked "honey-range" cover 1961–2017.

Series	ADF p	ADF: stationary (p<0.05)?	KPSS p	KPSS: stationary (p>0.05)?	Ljung-Box p (lag 10)
World temp	0.998	False	0.01	False	0.0
ΔWorld temp	0.0001	True	0.0417	False	0.0039
World colony	0.9885	False	0.01	False	0.0
ΔWorld colony	0.0318	True	0.0946	True	0.0
World honey	0.9977	False	0.01	False	0.0
ΔWorld honey	0.0	True	0.1	True	0.1291
Med temp (colony-range)	0.9899	False	0.01	False	0.0
ΔMed temp (colony-range)	0.0	True	0.1	True	0.0
Med colony	1.0	False	0.01	False	0.0
ΔMed colony	0.7348	False	0.01	False	0.0025
Med temp (honey-range)	0.9682	False	0.01	False	0.0
ΔMed temp (honey-range)	0.0	True	0.1	True	0.0
Med honey	0.9839	False	0.01	False	0.0
ΔMed honey	0.0	True	0.1	True	0.0317
Med efficiency	0.3132	False	0.01	False	0.0
ΔMed efficiency	0.0	True	0.1	True	0.0046

Table S2a. Panel coverage by country

Number of country-year observations, first and last reporting year for each of the 18 Mediterranean countries in the merged panel.

Country	n	First year	Last year
Albania	62	1961	2024
Algeria	54	1971	2024
Bosnia and Herzegovina	29	1996	2024
Croatia	26	1992	2017
Egypt	64	1961	2024
France	57	1961	2017
Greece	57	1961	2017
Israel	64	1961	2024
Italy	57	1961	2017
Lebanon	64	1961	2024
Libya	64	1961	2024
Montenegro	19	2006	2024
Morocco	64	1961	2024
Slovenia	26	1992	2017
Spain	57	1961	2017
Syrian Arab Republic	64	1961	2024
Tunisia	64	1961	2024

Türkiye	64	1961	2024

Table S2b. Panel coverage by year

Number of countries contributing to the merged panel in each year. Coverage rises from 13 countries in 1961 to 18 from the mid-2000s, and falls to 12 after 2017, when honey production value is no longer reported for Croatia, France, Greece, Italy, Slovenia and Spain.

Year	Number of countries	Year	Number of countries
1961	13	1993	16
1962	13	1994	16
1963	13	1995	16
1964	13	1996	17
1965	13	1997	17
1966	13	1998	17
1967	13	1999	17
1968	13	2000	17
1969	13	2001	17
1970	13	2002	17
1971	14	2003	17
1972	14	2004	17
1973	14	2005	17
1974	14	2006	18
1975	14	2007	18
1976	14	2008	18
1977	14	2009	18
1978	14	2010	18
1979	14	2011	18
1980	14	2012	18
1981	14	2013	18
1982	14	2014	18
1983	14	2015	18
1984	14	2016	18
1985	14	2017	18
1986	14	2018	12
1987	14	2019	12
1988	14	2020	12
1989	14	2021	12
1990	14	2022	12
1991	13	2023	12
1992	15	2024	12

Table S3. Leave-one-country-out sensitivity analysis

Basin-level Pearson correlation between temperature anomaly and registered colony number, computed with all 18 countries and with Türkiye excluded (1961–2023, level series).

Model	Pearson r	p
All 18 countries	0.896	<0.001
Excluding Türkiye	0.892	<0.001
Δr (impact of Türkiye)	0.004	

Table S4a. Country-specific HAC regression results — registered colony number

Country-specific regressions of registered colony number on temperature anomaly, estimated with Newey–West (HAC) standard errors. Raw coefficients are in colonies per 1 °C; standardized coefficients are obtained from z-scored variables and allow comparison

across countries. Countries with fewer than 20 country-years are excluded. DW = Durbin–Watson statistic of the classical OLS residuals; NW lag = Bartlett truncation lag used in the HAC estimator.

Country	n	Period	β (raw)	p (raw)	β (standardized)	p (standardized)	DW	NW lag
Albania	64	1961-2024	114,027	<0.001	0.767	<0.001	0.81	3
Algeria	64	1961-2024	118,123	<0.001	0.766	<0.001	1.1	3
Bosnia and Herzegovina	33	1992-2024	122,461	<0.001	0.621	<0.001	0.5	3
Croatia	32	1992-2023	191,654	<0.001	0.690	<0.001	0.72	3
Egypt	64	1961-2024	-49,698	0.448	-0.121	0.448	0.09	3
France	63	1961-2023	94,300	0.226	0.329	0.226	0.38	3
Greece	63	1961-2023	256,601	<0.001	0.714	<0.001	0.77	3
Israel	64	1961-2024	20,032	<0.001	0.743	<0.001	0.96	3
Italy	63	1961-2023	342,241	<0.001	0.846	<0.001	1.2	3
Lebanon	64	1961-2024	73,379	<0.001	0.662	<0.001	0.72	3
Libya	64	1961-2024	9,404	0.003	0.489	0.003	0.5	3
Morocco	64	1961-2024	-29,346	0.011	-0.329	0.011	0.13	3
Slovenia	32	1992-2023	20,652	0.335	0.235	0.335	0.28	3
Spain	63	1961-2023	955,455	<0.001	0.840	<0.001	1.21	3
Syrian Arab Republic	64	1961-2024	162,670	<0.001	0.719	<0.001	0.91	3
Tunisia	64	1961-2024	229,316	<0.001	0.802	<0.001	1.05	3
Türkiye	64	1961-2024	1,906,787	<0.001	0.719	<0.001	1.08	3

Table S4b. Country-specific HAC regression results — constant-price honey production value

Country-specific regressions of constant-price honey production value on temperature anomaly, estimated with Newey–West (HAC) standard errors. Raw coefficients are in thousand international dollars per 1 °C. DW = Durbin–Watson statistic of the classical OLS residuals; NW lag = Bartlett truncation lag used in the HAC estimator.

Country	n	Period	β (raw)	p (raw)	β (standardized)	p (standardized)	DW	NW lag
Albania	62	1961-2024	5,887	<0.001	0.832	<0.001	1.22	3
Algeria	54	1971-2024	6,042	<0.001	0.645	<0.001	0.58	3
Bosnia and Herzegovina	29	1996-2024	3,506	<0.001	0.572	<0.001	1.02	3
Croatia	26	1992-2017	10,172	0.009	0.553	0.009	0.95	2
Egypt	64	1961-2024	-5,442	0.002	-0.421	0.002	0.26	3
France	57	1961-2017	8,937	<0.001	0.501	<0.001	0.97	3
Greece	57	1961-2017	12,657	<0.001	0.529	<0.001	0.76	3
Israel	64	1961-2024	2,260	<0.001	0.587	<0.001	1.08	3
Italy	57	1961-2017	8,641	<0.001	0.630	<0.001	0.67	3
Lebanon	64	1961-2024	1,373	<0.001	0.502	<0.001	0.34	3
Libya	64	1961-2024	995	<0.001	0.554	<0.001	0.48	3
Morocco	64	1961-2024	4,103	0.027	0.504	0.027	0.64	3
Slovenia	26	1992-2017	-486	0.482	-0.156	0.482	2.21	2
Spain	57	1961-2017	41,144	<0.001	0.771	<0.001	1.1	3
Syrian Arab Republic	64	1961-2024	3,485	<0.001	0.748	<0.001	1.19	3
Tunisia	64	1961-2024	4,867	<0.001	0.775	<0.001	1.31	3
Türkiye	64	1961-2024	91,696	<0.001	0.648	<0.001	0.9	3

Table S4c. Country-specific HAC regression results — normalized honey production value per colony index

Country-specific regressions of the normalized honey production value per colony index on temperature anomaly, estimated with Newey–West (HAC) standard errors. Raw coefficients depend on each country's normalization baseline and are not directly comparable across countries; standardized coefficients should be used for comparison. DW = Durbin–Watson statistic of the classical OLS residuals; NW lag = Bartlett truncation lag used in the HAC estimator.

Country	n	Period	β (raw)	p (raw)	β (standardized)	p (standardized)	DW	NW lag
Albania	62	1961-2024	0.755	<0.001	0.654	<0.001	0.78	3
Algeria	54	1971-2024	0.308	<0.001	0.487	<0.001	0.46	3
Bosnia and Herzegovina	29	1996-2024	-0.042	0.406	-0.171	0.406	1.56	3
Croatia	26	1992-2017	-0.036	0.708	-0.067	0.708	0.82	2
Egypt	64	1961-2024	-0.335	<0.001	-0.558	<0.001	0.52	3
France	57	1961-2017	0.374	<0.001	0.675	<0.001	1.36	3
Greece	57	1961-2017	0.145	0.005	0.373	0.005	1.12	3
Israel	64	1961-2024	0.002	0.956	0.005	0.956	0.87	3
Italy	57	1961-2017	0.023	0.734	0.080	0.734	0.37	3
Lebanon	64	1961-2024	0.171	0.453	0.121	0.453	0.19	3
Libya	64	1961-2024	0.197	<0.001	0.500	<0.001	0.6	3
Morocco	64	1961-2024	0.627	0.008	0.567	0.008	0.57	3
Slovenia	26	1992-2017	0.113	0.620	0.083	0.620	1.23	2
Spain	57	1961-2017	-0.119	<0.001	-0.544	<0.001	0.81	3
Syrian Arab Republic	64	1961-2024	0.339	0.003	0.459	0.003	0.97	3
Tunisia	64	1961-2024	-0.008	0.926	-0.018	0.926	0.18	3
Türkiye	64	1961-2024	0.130	0.211	0.179	0.211	0.21	3

Table S5. Level-series and first-differenced correlations

Pearson (r) and Spearman (ϱ) correlations between temperature anomaly and each beekeeping indicator, computed on the level series and on the first-differenced (year-to-year change) series. Confidence intervals are moving-block bootstrap 95% percentile intervals (block length ≈15% of n, 2000 replicates). World series cover 1961–2024; Mediterranean colony series cover 1961–2023 and Mediterranean honey series 1961–2017.

Series	r (level)	95% CI (level)	ϱ (level)	r (differenced)	95% CI (differenced)	ϱ (differenced)
World — Temperature vs colonies	0.916	[0.68,0.94]	0.906	0.095	[-0.13,0.27]	0.074
World — Temperature vs honey value	0.939	[0.79,0.96]	0.937	0.292	[-0.06,0.55]	0.261
Mediterranean — Temperature vs colonies	0.896	[0.66,0.95]	0.844	0.141	[-0.07,0.28]	0.156
Mediterranean — Temperature vs honey value	0.809	[0.45,0.90]	0.765	-0.447	[-0.62,-0.27]	-0.438

Data and code availability

The processed analytical datasets (Mediterranean country-year panel and World series) and the analysis scripts used to generate all tables and figures are openly available in the repository cited in the Data Availability Statement of the main article. The underlying raw data are publicly available from FAOSTAT; the domains, item and element codes, and the extraction date are specified in Section 2.1 of the main article.
